# MAVENs: Motion analysis and visualization of elastic networks and structural ensembles

**DOI:** 10.1186/1471-2105-12-264

**Published:** 2011-06-28

**Authors:** Michael T Zimmermann, Andrzej Kloczkowski, Robert L Jernigan

**Affiliations:** 1 L. H. Baker Center for Bioinformatics and Biological Statistics, Iowa State University, Ames, IA 50011, USA; 2 Department of Biochemistry, Biophysics and Molecular Biology, Iowa State University, Ames, IA 50011, USA; 3 Bioinformatics and Computational Biology Program, Iowa State University, Ames, IA 50011, USA; 4 Battelle Center for Mathematical Medicine, The Research Institute at Nationwide Children's Hospital, Columbus, OH 43205, USA; 5 Department of Pediatrics, The Ohio State University College of Medicine, Columbus, OH 43205, USA

## Abstract

**Background:**

The ability to generate, visualize, and analyze motions of biomolecules has made a significant impact upon modern biology. Molecular Dynamics has gained substantial use, but remains computationally demanding and difficult to setup for many biologists. Elastic network models (ENMs) are an alternative and have been shown to generate the dominant equilibrium motions of biomolecules quickly and efficiently. These dominant motions have been shown to be functionally relevant and also to indicate the likely direction of conformational changes. Most structures have a small number of dominant motions. Comparing computed motions to the structure's conformational ensemble derived from a collection of static structures or frames from an MD trajectory is an important way to understand functional motions as well as evaluate the models. Modes of motion computed from ENMs can be visualized to gain functional and mechanistic understanding and to compute useful quantities such as average positional fluctuations, internal distance changes, collectiveness of motions, and directional correlations within the structure.

**Results:**

Our new software, MAVEN, aims to bring ENMs and their analysis to a broader audience by integrating methods for their generation and analysis into a user friendly environment that automates many of the steps. Models can be constructed from raw PDB files or density maps, using all available atomic coordinates or by employing various coarse-graining procedures. Visualization can be performed either with our software or exported to molecular viewers. Mixed resolution models allow one to study atomic effects on the system while retaining much of the computational speed of the coarse-grained ENMs. Analysis options are available to further aid the user in understanding the computed motions and their importance for its function.

**Conclusion:**

MAVEN has been developed to simplify ENM generation, allow for diverse models to be used, and facilitate useful analyses, all on the same platform. This represents an integrated approach that incorporates all four levels of the modeling process - generation, evaluation, analysis, visualization - and also brings to bear multiple ENM types. The intension is to provide a versatile modular suite of programs to a broader audience. MAVEN is available for download at http://maven.sourceforge.net.

## Background

One of the first dynamic computations of a protein was published by Levitt and Warshel in 1975 [1], a folding a coarse-grained peptide chain. The first publication widely recognized as a Molecular Dynamics (MD) simulation came two years later [2] and presented a simulation of the 56 residue bovine pancreatic trypsin inhibitor. Today's most advanced simulations, such as the fully atomic model simulation of the ribosome [3], (2.6 million atoms for a period of 10^6 ^CPU hours) represent significant improvements in computational technology and advance our understanding of the behavior of molecular systems.

There is growing evidence supporting the collectiveness of the motions of biomolecules [4-8]. Atomic MD takes into account the detailed modeling of the intrinsic randomness of the short time-scale motions. While true to the underlying atomic theories, this level of randomness may be distracting when analyzing motions of biomolecules on the longer time scale. To overcome such randomness, methods have been developed to determine the dominant motions within the trajectory - Principal Component Analysis (PCA) based on average covariances across the trajectory or Essential Dynamics [9]. The results of PCA have been shown to agree well with ANM modes [10,11]. The rigor of molecular dynamics simulations makes them ideal for investigating specific events, but less applicable for extracting mechanisms, and overly detailed for general purposes.

Normal Mode Analysis (NMA) using Elastic Network Models (ENMs) shifts the focus from simulating the motion of all atoms based on a detailed empirical force field to the harmonic motions of a set of springs and masses representing the starting structure. ENM is also an analytic solution yielding a basis set of orthogonal independent motions, rather than a simulation over time. The differences between various ENM types specify how the masses (computed from atomic coordinates or density contours) and springs (their harmonic interactions) are assigned or modeled; the exact chemistry of the object is not usually considered. Despite their lack of detailed chemistry, ENMs have proven themselves. Yang, et al. [10] as well as Bakan and Bahar [11] find that the motions computed using ENMs correspond well to the principal components of MD trajectories as well as to the spatial variance observed when multiple crystallographically determined structures of the same protein are superimposed or taken from an NMR ensemble. Sen et al. [12] emphasized the cooperativity of protein motions and how atomic detail is not necessary, while Yang, et. al. [13] shows the ability of elastic models to handle proteins across a broad range of sizes. From Jernigan, et. al. [14] we learn that functionally important motions of biomolecules are often governed by packing density: the basis for ENMs. Lu and Ma show that shape also plays an important role [15]. Early studies by Gō and Scheraga computationally defined the difference between local and collective motions [16], while Gō, Noguti, and Nishikawa demonstrated how the low frequency harmonic vibrations in proteins can be computed [17]. From these and other studies, it is becoming increasingly evident that molecular structures exhibit collective motions and that these motions can be sampled well by using ENMs. Methods that include distance dependent springs [18-20] or torsional angles [21-23] show improvements over the uniform springs and are also implemented in our software.

ENMs are capable of computing the important motions of biomolecules on time scales beyond the usual reach of atomic MD, and do so efficiently. Generation for small and medium sized proteins takes only seconds or minutes on a standard desktop computer, while typical MD studies require days or weeks of computer time on high performance systems or clusters. The largest molecular assemblages may require more time, but they can be further coarse-grained without loss of the major motions [24], once again making the computation of the dominant motions tractable. It is also possible to more efficiently solve numerically for only a subset of the normal modes. The low frequency modes contribute much more to the total motion than do the high frequency modes. Thus, many of the high frequency modes can be ignored without loss of important information. Detailed analyses can be achieved with elastic models by employing mixed-resolution models where most of the structure is coarse-grained but regions of special interest remain at atomic detail. Even with the proven efficiencies GPU computing brings to MD, the simplicity and effectiveness of NMA using ENMs will ensure their continued use. We aim here to make them more accessible.

## Implementation

**Figure 1:**
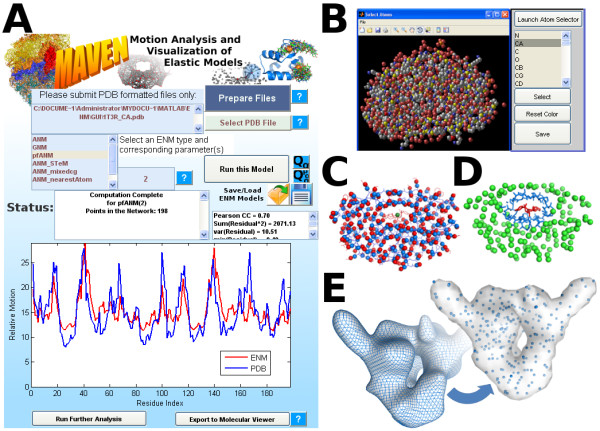
**MAVEN Interface Overview and Model Generation**. A) Screenshot of MAVEN having completed an ANM model of the HIV-1 protease 1T3R using 198 alpha carbons and  weighted springs. Motilities of each residue are shown; B-factors from the PDB file (blue) and mean squared fluctuations computed from the ENM (red). The correlation coefficient for these two curves is 0.70. B) Selecting by atom type within the Prepare Files module is shown with all atoms from 1T3R displayed. Methods to generate other coarse grained systems are provided and explained in the User's Manual including the average sugar and base position in nucleotides or C) residue or side chain centroid positions. D) Mixed resolution systems are also supported. We show the protease inhibitor in red and surrounding atoms as blue sticks with the rest of the structure coarse-grained to C^α ^atoms shown as green spheres. E) MAVEN has the unique ability to convert electron density maps into coarse-grained points. EMDB structure 1800 is shown as a density contour followed by an approximate surface filled with spherically coarse-grained points. All of these examples are given in greater detail in the User's Manual.

Here we present a platform for Motion Analysis and Visualization of Elastic Networks and Structural Ensembles (MAVEN), licensed under the Lesser General Public License so that MAVEN is freely available. Figure 1 provides a brief overview of the MAVEN interface and some of its features. It is built using MATLAB^® ^(2010a, The MathWorks, Natick, MA) and compiled into a standalone application, which can be run by the MATLAB Component Runtime (MCR). The MCR is freely provided from MathWorks and is distributed with MAVEN. Thus, MATLAB is not needed to run our application. The source code contains numerous self-documented MATLAB functions, which can be used without modification to extend other user's programs. Certain functions have been written in Perl or C++ to improve performance. MAVEN, its source code, MCR, User's Guide, and video tutorials are available on our website.

### Elastic Network Model

Protein structures are often represented as a coarse-grained elastic network by connecting C^α ^atom coordinates with harmonic springs. The Gaussian Network Model (GNM) is used to compute relative magnitudes of motion. It was originally proposed in 1997 [25,26], and employed Tirion's postulate [27] that all atomic contacts in proteins can be represented by a universal spring. The Anisotropic Network Model (ANM) proposed in [28], extends the GNM to also include directions of motion. ANM model generation consists of choosing which spatial points to describe the geometry of the system, construction of the Laplacian (or Kirchhoff) matrix using Equation 1, followed by eigen-decomposition of the second derivatives of the potential energy (see [28] for details). The eigenvectors are called normal mode shapes and eigenvalues their square frequencies. In Equation 1, a spring with stiffness γ is placed between atoms *i *and *j *if the distance between them, *d_ij_*, is less than the interaction cutoff radius, *r_c_*, (typically 10-13Å for ANM and 7.3 for GNM). The first six modes (one in GNM) correspond to rigid body rotation and translation of the entire system. Low frequency normal modes represent the collective motions of the system and have been shown to be biologically relevant. Higher modes display local motion that is less likely to be representative of functional motions of the biological system, but may be useful in identifying nodes that are important for energy transfer through the structure.(1)

We compute mean squared fluctuations of the structure using Equation 2. When *i *= *j*,  (example in Figure 1A). Changes in internal distance can also be calculated from Γ^-1 ^using equation 3. Because Γ is not invertible, we compute the pseudo-inverse,  where the summation of normal modes,*Q_i _*, and square frequencies, *λ_i _*(the eigenvector and eigenvalues, respectively), is over all relevant modes ({7 ... 3*N*} in ANM and {2 ... *N*} in GNM).(2)(3)

ENMs can also be constructed by considering all pairs of points to be in contact and their interaction strength (the spring constant) to be a function of their separation. This is implemented as an inverse power dependence as in equation 4.(4)

Many other model types have been developed including methods where bond or dihedral angles are explicitly taken into account [21-23] and are implemented in MAVEN by incorporation of the freely available Spring Tensor Model developed by Lin and Song [21]. We also implement the nearest neighbor method which utilizes a coarse-grained model (usually one point per residue), but assigns contacts between residues based on an atomic model of the system. To facilitate detailed analysis, one may employ mixed resolution modeling [29,30]. In this scheme most of the structure is coarse-grained, but any region of interest is included in greater detail (see Figure 1D). One may then choose different cutoff values for the two regions and use the geometric mean of the cutoff values as the cutoff for connections between them [29].

### Comparing ENMs to Ensembles of Structures

**Figure 2:**
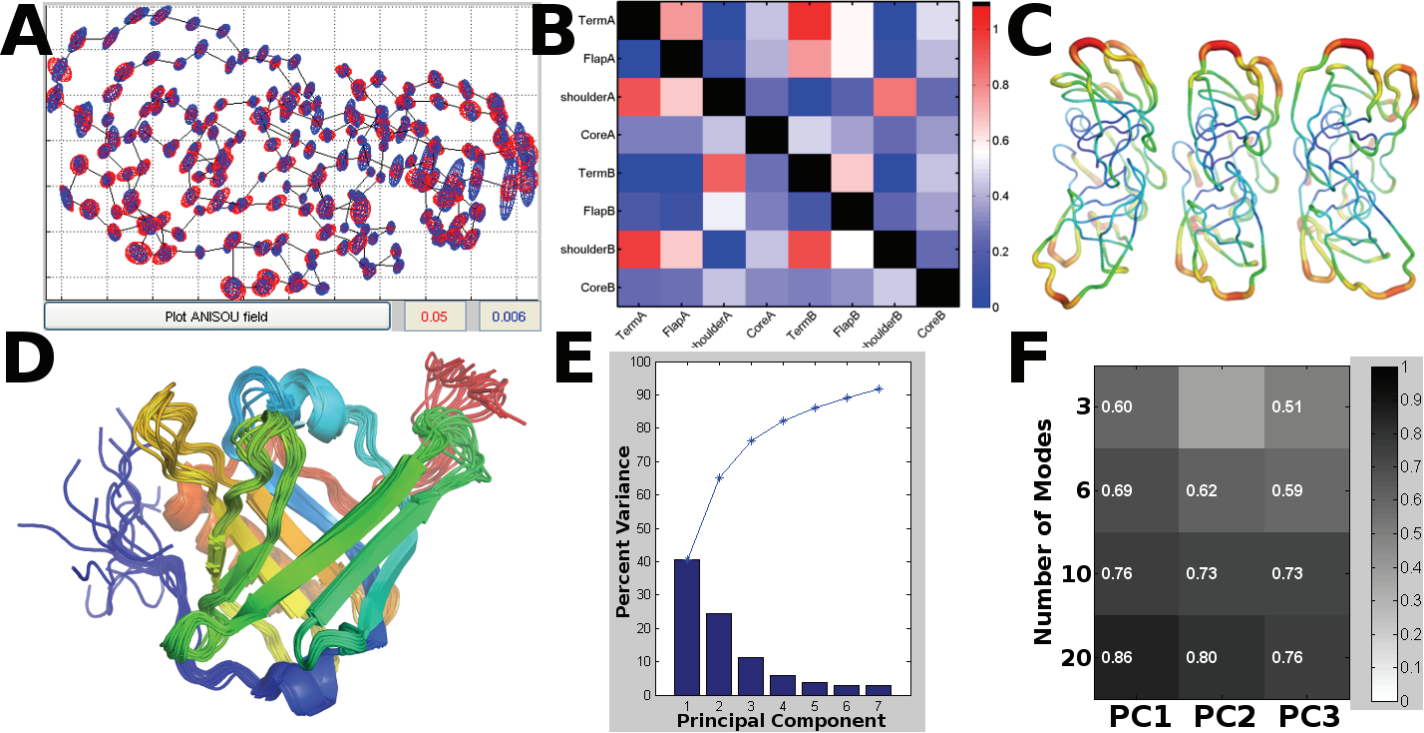
**Analysis Features of MAVEN**. A) Within MAVEN, we plot the anisotropic displacement tensors from PDB file 1T3R in red and computed from  weighted ANM in blue. B) How strongly the directions of motion of eight parts of the protease structure are correlated with one another in the first mode of motion is summarized. C) We display three frames in a top-down view of the animation of mode 1 that was exported to PyMOL: the negative mode direction (left), the initial structure (center), and the positive mode deformation (right). Coloring and tube thickness is by B-factor. D) Visualization of the NMR ensemble described in PDB file 2KTD in PyMOL. Coloring is blue to red from the N to C-terminus. E) Principal Component Analysis of the ensemble is performed and the variance in each PC and the cumulative variance plotted. F) Computation of the cumulative overlap between a set of low frequency modes generated from the first member of the ensemble and the first 3 PCs, using equation 6. MAVEN always displays a heatmap, but only displays text for significant relationships (CO > 0.5).

Principal Component Analysis of a set of structures can be used to describe the functional ensemble of the biomolecule. This is performed in MAVEN by computing the covariance matrix for atomic positions within the ensemble and applying singular value decomposition to it. The result is a set of vectors that capture the ensemble variance as efficiently as possible; the first PC explains the most variance possible by a linear approximation, the second as much of the remaining variance as possible, and so on (Figure 2E). These vectors can be compared to the normal modes from an ANM model using equations 5-7 which describe the Overlap between the *i^th ^*PC (*P_i_*) and the *j^th ^*mode (*M_j_*), Cumulative Overlap between the first *k *normal modes and the *i^th ^*PC (Figure 2F), and overlap between the space spanned by first *I *PCs and the first *J *low frequency modes (their Root Mean Square Inner Product), respectively, and are further described by Tama and Sanejouand [31] and Leo-Macias, et. al. [32].(5)(6)(7)

### Model Generation

Perhaps the most important step in ENM modeling is choosing which points will represent the system. Originally, ENMs were proposed for use with all atom coordinates [27], but when it was learned that nearly the same motions are obtained with coarse-grained structures [12,24], this became the more common approach. Within MAVEN, one may retain all atoms, choose standard representations like C^α ^atoms, pick specific atom types, or generate centroid points from residues, side-chains, or bases (for examples see Figure 1). A set of points can be further coarse-grained using spherical coarse-graining. This task is accomplished by selecting an initial point (or set of points) that will be retained. All points within a given radius of the retained point will be removed from the model. The closest point that was not removed is then added to the retained set. This process continues until no more points can be removed. The result is a spatially more uniform distribution of points than one would likely have after selecting points linearly along the sequence. Finally, MAVEN allows the user to employ low resolution density maps (Figure 1E); a data source that has rarely been used with these methods. Since packing density and shape are the properties most critical to ENMs, model points picked along the desired density contour should provide a reasonable coarse-graining. Doruker and Jernigan have shown that similar motions are extracted from proteins and from the protein's molecular volume filled with lattice points [33], further showing the potential usefulness of density maps in ENM modeling. This method represents an extension to understand the dynamics of very large structures where there are no atomic coordinates.

## Discussion

Because of the usefulness of the ENM method, web and standalone applications have been constructed [23,34-38]. Web servers have the advantage of near universal accessibility, but often lack flexibility and extensibility. Existing standalone applications tend to only implement one ENM type and often force the user to use one representation, for instance alpha carbons only, thereby preventing the use of nucleotides, sugars, or small molecules. In developing MAVEN we seek to incorporate many ENM methods including support for dihedral angle and mixed resolution modeling, as well as to facilitate model generation and shape based coarse-graining (see Methods).

The first major feature of this platform is the ability to construct many types of ENMs whereas other servers and applications available are restricted to one or two types. These include the standard cutoff based models, distance dependent springs, nearest neighbor, Spring Tensor [21], and mixed resolution. The nearest neighbor method generates a coarse-grained model, but uses an atomic model for determining connectivity. The Spring Tensor model expands the energy function of ENMs to account for bond and torsion angle changes. Mixed coarse-graining represents a compromise: part of the system remains in high detail with the remainder coarse-grained. With mixed resolution, one is able to analyze molecular effects on motions such as chemical modifications, mutations, drug binding, proline isomerization, or post-translational modifications, while retaining nearly the computational efficiency of coarse-grained NMA. A second feature of this application is the ability to handle large systems through sparse matrix methods and the ability to calculate only a set of the lowest frequency modes. Since the contribution of each mode to the total motion decreases quickly, calculating only the lowest frequency modes captures the majority of dynamics while requiring considerably less computer resources. A further benefit of MAVEN is that it is setup to accept protein, RNA, DNA, and small molecule coordinates. From an unprocessed PDB file, one can generate a standard alpha carbon model, our atom selector can be used to save a subset of atom types for use in any ENM, points can be picked from electron density contours, united atoms representing the centroid of a set of atoms can be generated, or one may compose or edit an initial model using other software (such as a molecular viewer) and use MAVEN for ENM generation and analysis. See Figure 1 for examples of these model types.

Multiple analysis features are presently included. Selected methods are shown in Figure 2. These include the ability to analyze Principal Components constructed from multiple static structures, an NMR ensemble, or frames from an MD trajectory and compare them to the normal modes. Multiple studies including [10] and [11] have shown that the variance seen in ensembles of structures derived from MD trajectories, NMR, or X-ray crystallography can be reproduced with ENMs. This represents an important method for ENM model validation and further exploration of functional motions. MAVEN also has the ability to compare two ENMs of different types or having different parameter choices, and to analyze the effect of the mode-motions on subsets of the structure, comparing within or across subsets. For a full list of our analysis features and examples of their use, please consult our user's guide (Additional File 1). Future additions are likely to include automated methods for batch model generation and comparison, spectral analysis, or Markov Propagation Model simulations which probe paths of information transfer within the structure and were recently cast into the ENM framework [39].

Analysis and visualization of the resulting data can be performed within MAVEN. Alternatively, PyMOL [40], VMD [41], and other molecular viewers specialized for visualization of molecular systems and can be used. For this reason, animations of the modes are saved in PDB file format (each frame is a separate MODEL) so that any molecular viewer can be used to visualize them. The MAVEN interface is configured so that generation of animation files, loading them into a molecular viewer (PyMOL has been our preferred viewer), and setting up an appropriate initial view is performed.

## Conclusions

MAVEN implements multiple types of ENMs for atomic, coarse-grained, and mixed coarse-grained representations and assists the user in generating these, permitting selection by atom or residue type, spherical coarse-graining, and united atom modeling (combining multiple atoms into one placed at their mean position). By implementing these and other methods for ENM model generation, MAVEN allows for diverse and detailed hypothesis testing. One may use sparse methods for fast mode generation, making large systems more tractable. Analysis of internal motions, directional correlations within the structure, comparing the mode shapes to the variance within a structural ensemble, and comparing anisotropies of motion are presently included. MAVEN, source code, and all optional components are freely available to assist the scientific community with dynamic studies of biomolecules.

## Competing interests

The authors declare that they have no competing interests.

## Availability and requirements

Project name: MAVEN

Project home page: http://maven.sourceforge.net

Operating system(s): Windows, Mac, and Linux

Programming language: MATLAB, Perl, and C++

Other requirements: MCR (freely available on our web site)

License: Lesser General Public License (LGPL)

## Authors' contributions

MTZ wrote and tested the application, and wrote the manuscript and Users Guide. AK helped develop MAVEN's goals and provided algorithm guidance. RLJ conceived the study, participated in its design and testing, and wrote the manuscript and Users Guide. All authors read and approved the final manuscript.

## Supplementary Material

Additional file 1 **MAVEN 1.0 User's Guide**. The User's Guide includes more details describing the features of MAVEN, algorithms used, and examples of use. It is available along with MAVEN distributions and is included here to provide readers with a full description of the capabilities of MAVEN. Click here for file
