# Supplementary material for: MAVENs: Motion analysis and visualization of elastic networks and structural ensembles
Source: BMC Bioinformatics. 2011 Jun 28;12:264. doi: 10.1186/1471-2105-12-264 (PMC3213244; doi:10.1186/1471-2105-12-264)
Supplement: Additional file 1 — MAVEN 1.0 User's Guide. The User's Guide includes more details describing the features of MAVEN, algorithms used, and examples of use. It is available along with MAVEN distributions and is included here to provide readers with a full description of the capabilities of MAVEN. [file 1471-2105-12-264-S1.PDF]

MAVEN: Motion Analysis and Visualization of Elastic Networks and Structure Ensembles

Version 1.1 User's Manual

Contents:

1. Overview
   1.1. What is MAVEN?
   1.2. Major Features
      - Parsing structure files
      - Generating an ENM
      - Analysis of the ENM
      - Exporting and Visualization
   1.3. Interface Overview
   1.4. System Requirements
2. Model Generation (Prepare Files)
   2.1. Selecting and reading a structure file
   2.2. Using the atom type selector
   2.3. Uniform coarse-graining
      2.3.1. Alpha carbon models of proteins
      2.3.2. Generate centroid positions (United Atoms) of proteins or nucleic acids
      2.3.3. Reading electron density maps or image files and converting them to PDB files
      2.3.4. Spherical coarse-graining
         → iteratively remove points that are within a cutoff distance of one another
   2.4. Mixed resolution modeling (see 3.2.6 for running this type of model)
3. Running an ENM
   3.1. Quick ENM buttons that automatically parse coarse-grained models from unprocessed files
   3.2. Methods and parameter inputs for each ENM type
      3.2.1. GNM → Gaussian Network Model
      3.2.2. ANM → Anisotropic Network Model
      3.2.3. Distance dependent springs
         → Connect all pairs of points and scale their interaction strength by a power of their separation
      3.2.4. STeM → An ENM which includes sequential bond lengths, angles, dihedral angles, and nonbonded terms
      3.2.5. Nearest Atom
         → Make a coarse-grained model where spring connections are assigned based on an all atom representation
      3.2.6. Mixed Resolution (see 2.4 for making the input PDB file)
4. Analysis
   4.1. B-factors and their correlation with computed fluctuations
   4.2. Calculating Anisotropy of motion and comparing to PDB ANISOU records

4.3. Fluctuations in internal distances
4.4. Correlated Motion between two parts of the structure
4.5. Compare one ENM to another
4.6. Overlap Matrices → Correlated motion within the structure
4.7. Compare modes to Principal Components of a structural ensemble

5. Exporting Data
5.1. Date to text file → ENM summary, B-factors, eigenvalues, and mode shapes
5.2. Molecular Viewers → how to visualize and animate the modes using PyMOL or VMD
5.3. Visualization Examples

6. References – Please also see the MAVEN publication in BMC Bioinformatics

1) Overview

The structures of biomolecules have become increasingly important for new biological discoveries. Tens of thousands of small to medium sized protein structures have been solved experimentally as well as a growing number of polynucleotide systems and some larger entities. New methods for structure determination of a wide range of biomolecules are under consideration across the world. A revolution in biology occurred (and is still being felt) due to the availability of molecular structures and structure determinations. This represents a true deepening of knowledge for biology. This was due to researchers being able to explore mechanisms, active sites, and other topological properties that were otherwise inaccessible.

Now that many static structures are available, much attention is focused on determining their dynamics. Elastic Network Modeling has become a popular method in biophysics and molecular biology for determining principal dynamics of biomolecules. It is a type of normal mode analysis where the structure is typically (but not necessarily) treated in a coarse-grained way and a simple energy function is employed. This may be as simple as the one parameter Gaussian Network Model (GNM) or more complex methods as in the Spring Tensor model where three and four body interactions (bond and torsion angles, respectively) are considered. Here we present MAVEN: a platform for generating and analyzing ENMs.

1.1) What is MAVEN?

MAVEN is a freely available standalone application for generating and analyzing Elastic Network Models (ENMs). It has been developed with the goal of bringing ENMs to a wider audience and easing the steps of model generation and analysis. MAVEN is freely available for download at (http://maven.sourceforge.net).

1.2) Major Features

A major feature of this platform is the ability to construct many types of ENMs whereas other servers and applications available are restricted to fewer types. These include the standard cutoff based models, distance dependent springs, nearest neighbor, Spring Tensor [11], and mixed resolution. The nearest neighbor method generates a coarse-grained model, but uses an atomic model for determining connectivity. The Spring Tensor model expands the energy function of ENMs to account for bond and torsion angle changes. Mixed coarse-graining represents a method for computing modes of motion in a coarse-grained system, while still being able to analyze molecular effects on those motions such as residue or base mutations, drug binding, proline isomerization, or post-translational modifications. A second feature of this application is the ability to handle large systems through sparse matrix methods and the ability to calculate only the lowest frequency modes. Since the contribution of each mode to the total motion decreases quickly, calculating only the lowest frequency modes captures the majority of dynamics while using considerably less computer resources. A further benefit of this platform is that it is setup to accept protein, RNA, DNA, and ligand coordinates. One can generate a standard alpha carbon model in only three clicks from an unprocessed PDB file, or our atom selector can be used to save a subset of atom types for use in any ENM, points can be picked from electron density contours, united atoms representing

the centroid of a set of atoms can be generated, or one may compile an initial model using other software (such as a molecular viewer) and use MAVEN for ENM generation and analysis.

1.3) Interface Overview

In this section we will introduce the MAVEN interface and explain many of its features. Figure 1.3 (Figure numbers correspond to section numbers) provides an annotated screenshot and points to which sections provide further explanation. Video tutorials are also available at the MAVEN web site (http://maven.sourceforge.net). MAVEN only accepts PDB formatted files (see www.rcsb.org). Data files of other types or formats will not be read properly. Density maps can be used by converting them within the Prepare Files module (see Section 2.3.3).

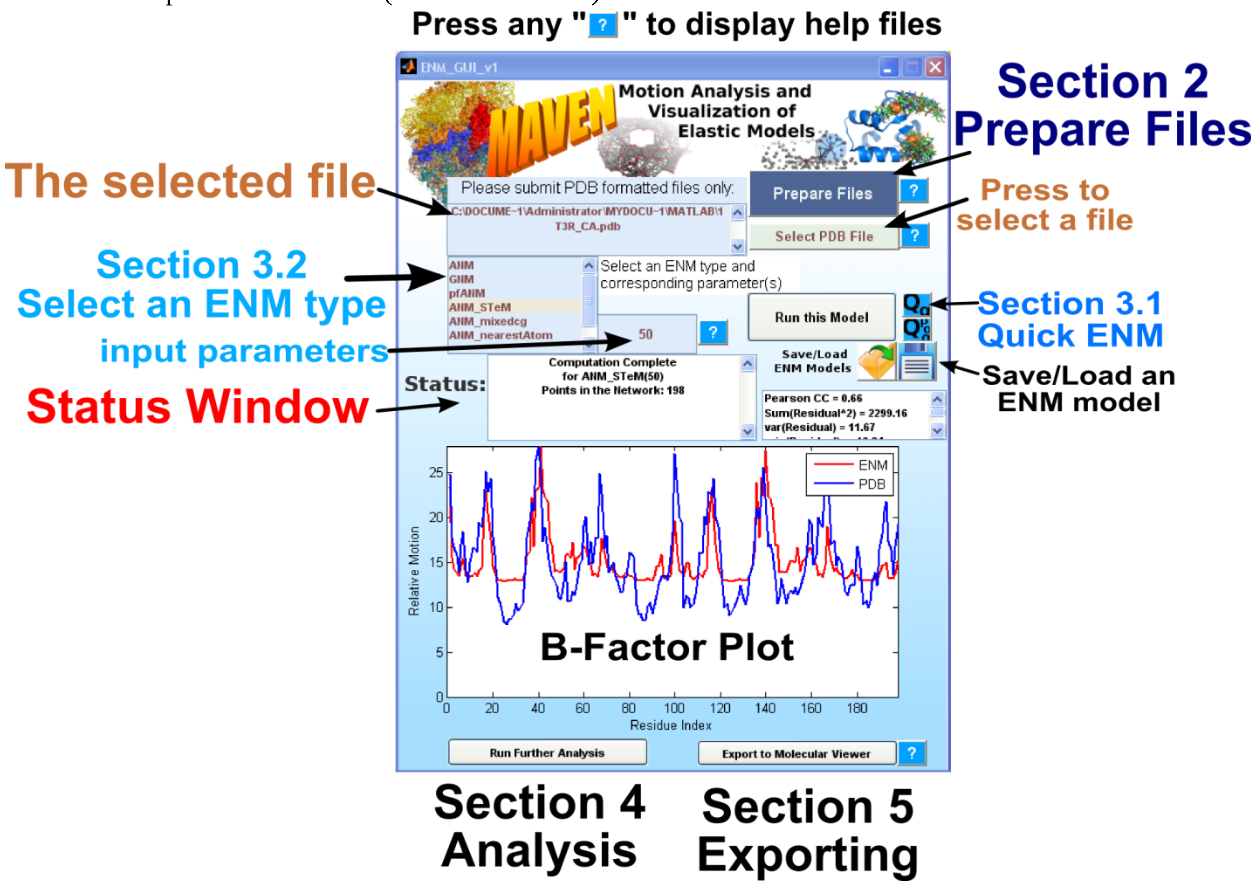

Figure 1.3 Interface Overview. The MAVEN interface is shown and annotated with the corresponding section number for further details about each component. Note that this User's Guide was generated using the Windows build of MAVEN. There may be some minor differences in the look of the interface on the other operating systems. The selected file is displayed. Throughout the model preparation, building, analysis, and exporting processes the Status Window will be continually updated to keep the user apprised of what MAVEN is doing. Results of computations are often displayed here as well.

1.4) System Requirements

MAVEN has been built independently on Windows, Macintosh, and Linux operating systems. Be sure to download the appropriate distribution for your system keeping in mind whether you have 32 bit or 64 bit

architecture. MAVEN is coded in a mixture of MATLAB, Perl, and C++. The MCR should be the only dependency required to run MAVEN, but a C compiler may be required to install the MCR. If your system does not meet the requirements for installing the MCR, the installation dialog will alert you as well as provide information on supported compilers. On Windows systems, this will likely be Microsoft Visual Studio Express, which is free in Windows. Linux and Mac systems should have gcc installed as part of the operating system.

Our source code is also made available with instructions for compiling or using the code outside of MAVEN. This provides users on unsupported OS/Architecture combinations access to MAVEN in addition to providing flexibility for extending MAVEN with user-made analysis routines. Source code written in MATLAB will require MATLAB to be used, but Perl and C++ code can be executed with appropriate compilers.

We do not recommend using MAVEN on a computer with less than 1GB of RAM unless only small models will be generated (<500 points). As the size of systems increases, MAVEN will require more memory. It is recommended to have at least 2 GB of RAM and a processor that operates at or above 2.5 GHz, but analysis of some structures may require more. If you run into memory barriers, most of the ENM functions have an optional parameter that is the number of requested modes. Requesting a smaller number of modes will decrease the required memory and computing time.

Disk Space

The MCR requires approximately 400 MB of disk space for use.
MAVEN requires only 1.5 MB of space. Documentation and examples total at worst 25 MB.

MAVEN depends on the MATLAB Component Runtime (MCR); a compiled library that interprets compiled MATLAB code. It is likely that the MCR system requirements are similar to the MATLAB system requirements (other than hard drive space – see below) which can be viewed, for all operating systems and architectures, at: http://www.mathworks.com/products/matlab/requirements.html.

2) Model Generation - Prepare Files

The "Prepare Files" module will help you to take a raw PDB formatted file and prepare it for computations. To learn more about PDB and the PDB file format, please see: www.rcsb.org. For information regarding the MRC electron density maps that are able to be used by MAVEN, please consult the Electron Microscopy Databank at www.emdatabank.org.

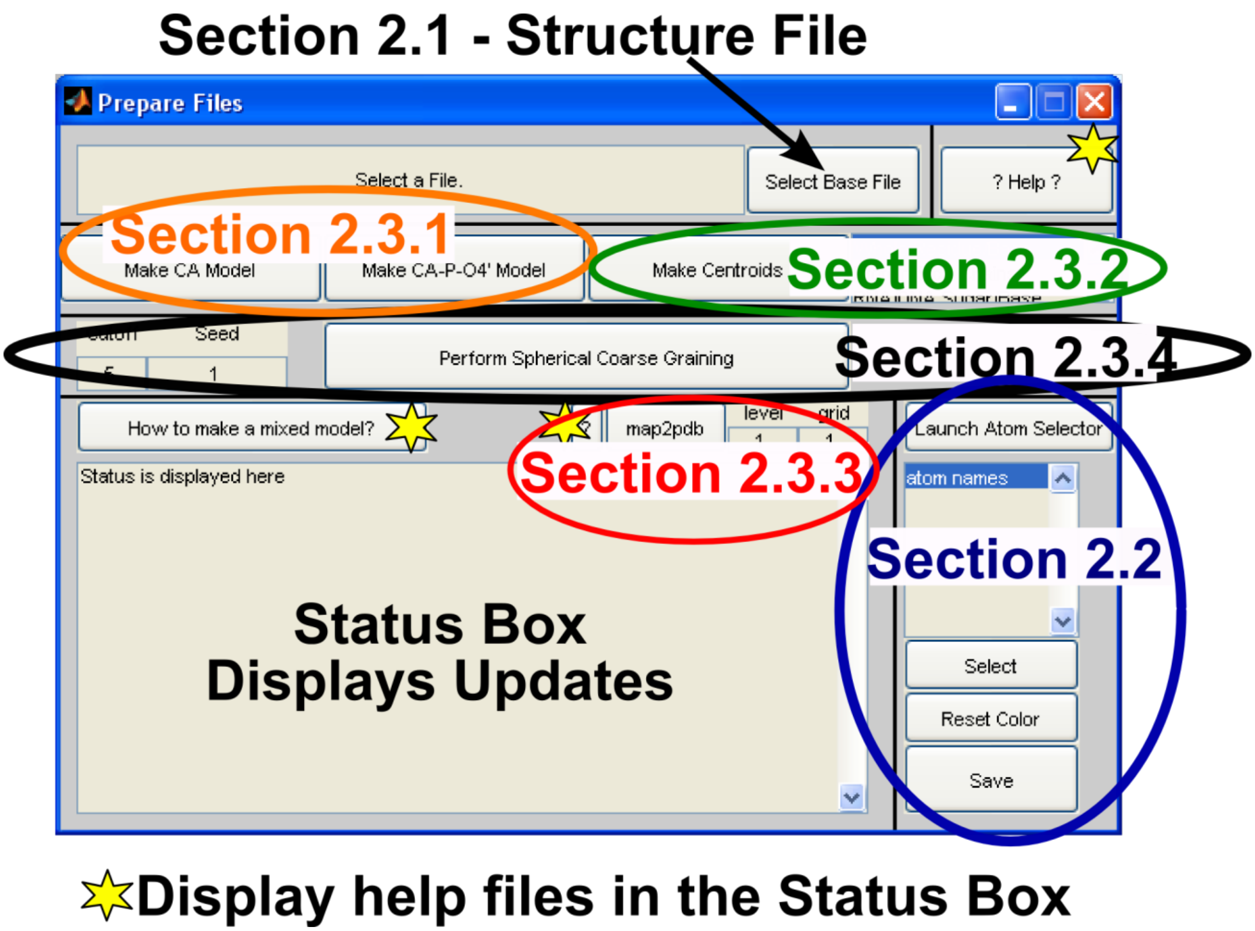

Figure 2.0 Prepare Files Module Overview. The figure above displays the Prepare Files module, annotated by which section further describes each part. From here, users can select atoms by type, perform spatial coarse-graining, convert density maps into PDB files, and more. Help files are accessible within MAVEN for each of the methods described here.

2.1) Selecting a Structure file

Within the Prepare Files module, you should first select a file (PDB or MRC density map) to work with. If a 4 character PDB structure ID is typed in the text box, then MAVEN will contact the PDB and retrieve the file. In this manual, this file will be referred to as the “structure file” or occasionally the “base file.” It may be a raw PDB file, or one that has been processed by other programs. As long as the PDB format is maintained the file will be read properly.

The first item to note is that PDB files contain headers that give a lot of information about the experiment that derived the given structure. The most critical information for MAVEN are the [x,y,z] coordinates of all atoms in the structure. Commonly, a model based upon the alpha carbons of proteins (see CA model in section 2.2 and/or 3.1), or the phosphate and/or O4' atoms of RNA and DNA backbones is used. Electron density maps can be imported into MAVEN by converting regions of density into coordinate points and saving those points as a PDB formatted file (see map2pdb in section 2.5).

The original file will not be modified by any of these scripts. New files will be placed in the same directory as the original structure file (base file). These processed files may be used for model generation via “Select PDB File” in the initial MAVEN window (see section 1.3). Note that the analysis (section 4) and export (section 5) modules assume that all relevant files (such as ANISOU files) are in the same directory as the structure file. This is the default behavior of the Prepare Files module.

Segment IDs are not part of the official PDB file format, but are supported by MAVEN. The official PDB file format does not accommodate structures with more than 99999 atoms. Molecular viewers such as VMD and PyMOL, as well as many other programs, have added the Segment ID (columns 73-76) to overcome this limitation. Each segment can include up to 99999 atoms.

2.2) Using the atom type selector

Launching the Atom Selector will read in the structure file, list all atom types in the structure file, and initialize a simple molecular viewer (this may take a few moments). Atom types can be selected by clicking on their names in the list (lower right of the Prepare Files module) and then pressing the [Select] button. You may select as many types as you wish by holding the [shift] and [ctrl] keys while clicking. Selected atom types are colored yellow in the viewer. Clicking the [Save] button will make a PDB file containing only the selected atom types (all atoms that have been colored yellow). To clear the atoms currently selected, use the [Reset Color] button.

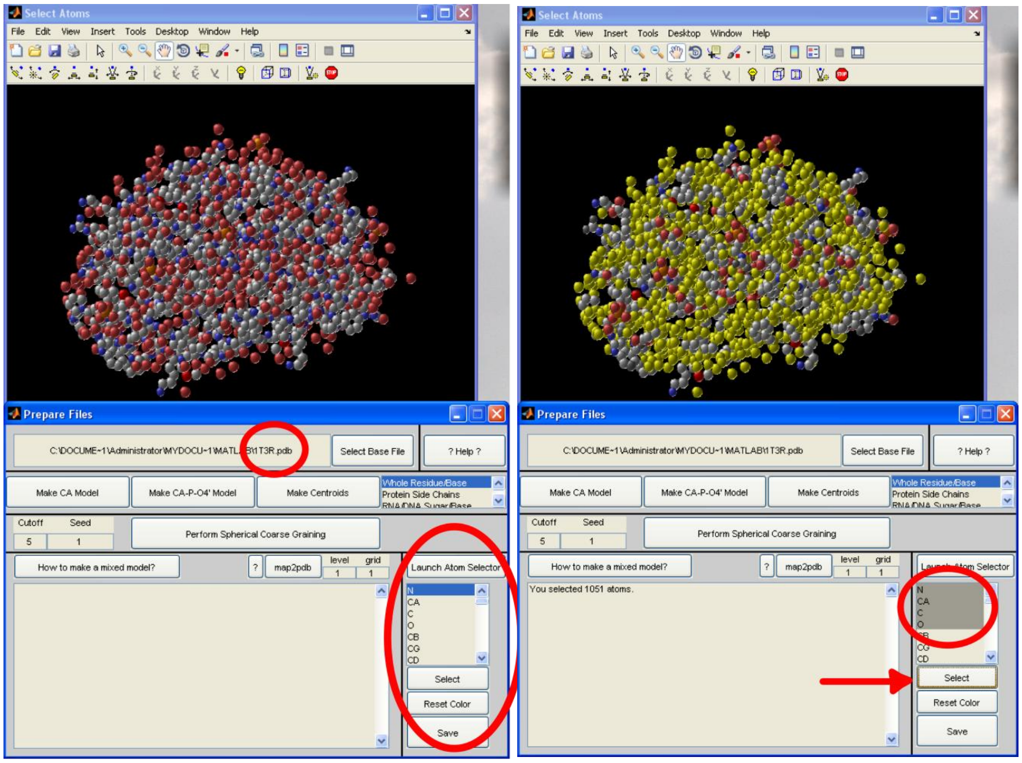

Figure 2.2 Example of selecting by atom type. To the left we show the PDB file 1T3R displayed in the Atom Selector. This view does not look exactly like the other images of this protease structure as all solvent atoms are also displayed. No atom types have been selected. After selecting atom names N, CA, C, and O (protein backbone atoms) and pressing the [select] button, the corresponding atoms are displayed in yellow and we are informed that 1051 atoms are selected. Pressing the [Save] button would make a PDB file consisting of only these selected atoms. The status window will display the name of the newly generated file.

2.3) Methods for Uniform Coarse-Graining

The next sections describe MAVEN's methods for uniform coarse-graining (cg). The main advantage of cg models is reduced computation time. It has been shown that the motions derived from coarse-grained proteins are very similar to those of atomic systems. As packing density and shape are the primary properties that ENMs depend on, it is important that the model points reflect that original density (spatial distribution of points) and shape of the structure.

2.3.1) alpha carbon models

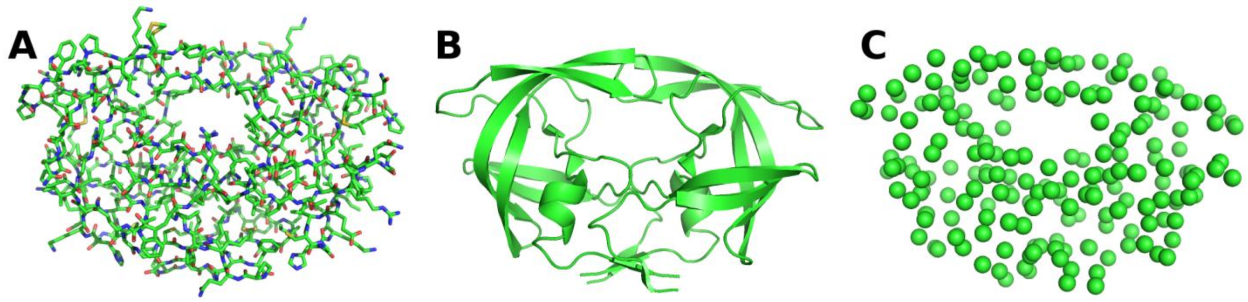

Figure 2.3.1 Examples of Protein Representations. A) PDB file 1T3R shown as "sticks." This level of detail does not show any solvent atoms or any of the system dynamics, yet it is complex. B) Structures are often shown in simpler "cartoon" views that emphasize the path of the peptide backbone through space. C) We show spheres for each C^{α} atom in the structure – a common way to represent proteins in coarse-grained ENMs.

Elastic Network Models consisting of alpha carbon (C^{α} atoms are given an atom type of "CA" in PDB files) atoms from proteins are the most common type. This one point per residue level of coarse-graining makes model generation fast and retains a one-to-one relationship with sequence information. Also, alpha carbons are part of the protein backbone, so their position informs also about the overall fold.

For RNA or DNA systems, the counterpart is a model based on the backbone phosphate atoms (PDB atom type "P"). Atoms from the sugar ring (most often "O4'" and occasionally "C2'") ought to be included so that the density of points is more similar to alpha carbon models of proteins, if both are included in a structure. Thus, nucleotide systems can be represented likewise in a coarse-grained way to preserve the backbone topology.

Please refer to section 3.1 for making these types of models quickly by bypassing the Prepare Files module.

2.3.2) Generate centroid positions (United Atoms)

It may be of use to choose model points that do not correspond exactly to atomic points. For this reason, we provide the capability to compute the centroid of a group of atoms. Possible selections include the centroid of each residue, amino acid side chain, or of each nucleotide sugar and base. Note that running this script on mixed DNA/RNA/protein systems may result in some inappropriate centroid atoms. For example, asking for the ribose centroid of a protein is not meaningful. If MAVEN generates a cg point,

the resulting centroid will not be for a ribose sugar ring (excluding the case of glycoproteins). Similarly, Nucleotide bases are not normally thought of as having side chains.

The following atom name lists are used for determining which group an atom is in:

```
nucleotide sugar   = c1' c2' o2' c3' o3' c4' o4' c5' o5'
nucleotide base    = n1 c2 o2 n2 n3 c4 n4 o4 c5 c6 n6 o6 n7 c8 n9
protein backbone   = n ca c o
protein side chain = not protein backbone
```

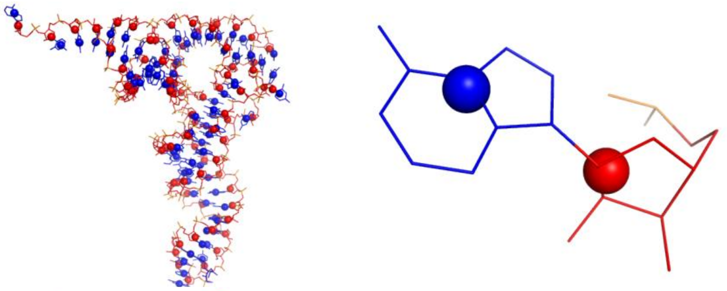

Figure 2.3.2*i* United Nucleotide Sugar and Base. tRNA structure 1TRA is shown in a lines representation with backbone and ribose rings colored red and the nucleotide base blue. United atoms are shown as spheres. A close up view is also given for one nucleotide.

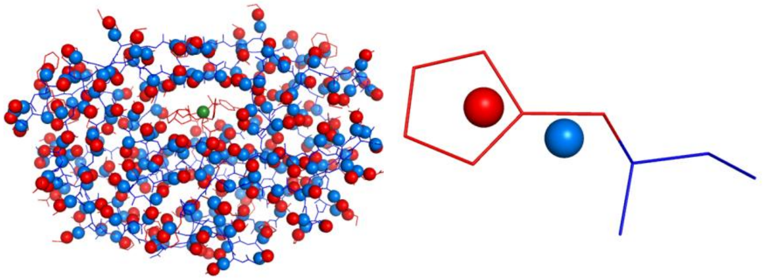

Figure 2.3.2*ii* United residues and side chains. Using the HIV-1 Protease 1T3R, united side chains are shown as red spheres and united residue positions in blue. Notice that united residues also generates a united atom point for the inhibitor molecule (green).

2.3.3) Reading electron density maps

The function “map2pdb” is designed to read in an MRC formatted 3D density map and convert all grid points that are at least "level" in value to a PDB file which contains a pseudo-atom for each density point. Thus, "level" is used as density contour. All grid points with an associated density value greater than or equal to "level" will be made into a pseudo-atom. The space between each grid point will be "grid" in Angstroms. If the density map is downloaded from a database like the EMDB (www.emdatabank.org), a suggested level will be given. See Figure 2.3.3*i* which uses EMDB structure 1800, the HIV-1 spike (http://emsearch.rutgers.edu/atlas/1800_mapparams.html).

It is notable that the functions used will work for any MRC volume or density file. Thus any type of tomographic reconstruction or image stack that is saved in the MRC file format can be analyzed using MAVEN.

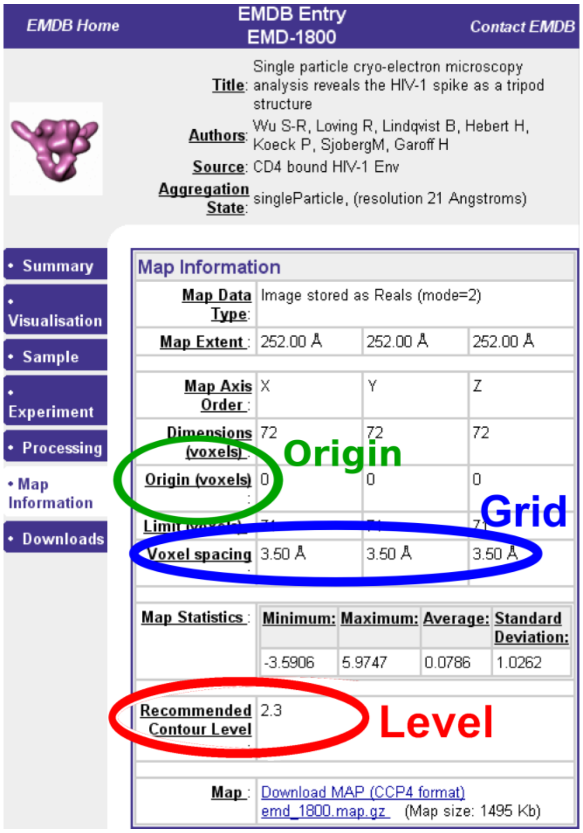

Figure 2.3.3*i* EMDB entry 1800 Map Information. From this panel we can see the recommended density contour as well as the grid spacing in the density map. This information is all the user needs to provide to convert the map into PDB file format. The origin determines the coordinates of the first data point (in voxel spacing).

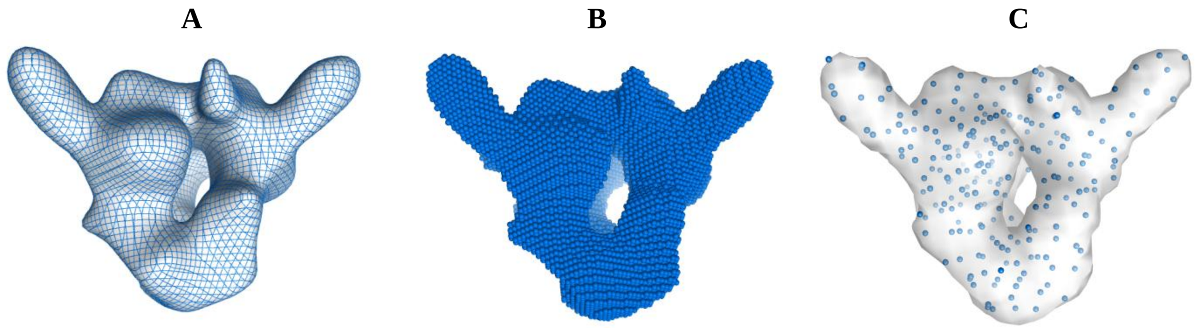

Figure 2.3.3*ii* Conversion from Density to coarse-grained pseudo-atoms. A) The map is visualized at the 2.3 density level. B) After selecting "EMD_1800.map" as the structure file within the Prepare Files module (this file is provided in the Examples folder in the MAVEN distribution), we set the Grid to 3.5 and Level to 2.3 and run map2pdb. The resulting PDB file is visualized as spheres. It is evident that many points are generated with this method and the representation is dense. C) The PDB file from B) is coarse-grained using spherical coarse-graining (see Section 2.3.4 for details). An approximate surface representation of the points in B) is shown for comparison. This more sparse representation will require a longer ENM cutoff, but will be much less demanding for calculations and still provide approximately the same motions.

We use readMRC.m which was downloaded from the MATLAB file exchange on Nov 9, 2010. The function is free for academic use as long as the following legal text is retained with its distribution:

readMRC
Copyright (c) 2010, Fred Sigworth
All rights reserved.
THIS SOFTWARE IS PROVIDED BY THE COPYRIGHT HOLDERS AND CONTRIBUTORS "AS IS" AND ANY EXPRESS OR IMPLIED WARRANTIES, INCLUDING, BUT NOT LIMITED TO, THE IMPLIED WARRANTIES OF MERCHANTABILITY AND FITNESS FOR A PARTICULAR PURPOSE ARE DISCLAIMED. IN NO EVENT SHALL THE COPYRIGHT OWNER OR CONTRIBUTORS BE LIABLE FOR ANY DIRECT, INDIRECT, INCIDENTAL, SPECIAL, EXEMPLARY, OR CONSEQUENTIAL DAMAGES (INCLUDING, BUT NOT LIMITED TO, PROCUREMENT OF SUBSTITUTE GOODS OR SERVICES; LOSS OF USE, DATA, OR PROFITS; OR BUSINESS INTERRUPTION) HOWEVER CAUSED AND ON ANY THEORY OF LIABILITY, WHETHER IN CONTRACT, STRICT LIABILITY, OR TORT (INCLUDING NEGLIGENCE OR OTHERWISE) ARISING IN ANY WAY OUT OF THE USE OF THIS SOFTWARE, EVEN IF ADVISED OF THE POSSIBILITY OF SUCH DAMAGE.

2.3.4) Spherical coarse-graining

For many systems, we only want to consider a subset of the points available to us. Typically, we choose alpha carbons since they represent the location of the peptide backbone and there is thus one point per amino acid. This level of coarse-graining provides a straightforward correspondence between the structure and sequence. Sometimes, either for a simpler view of the system, or for computational requirements, one may wish to further reduce the number of points considered. For this purpose, we provide a method to spherically coarse-grain a set of input points.

We begin with the seed point. This refers to the line number of the input file, if the file has been pre-processed. The coordinates in “Structure file” are looped through and all points within “Cutoff” of the seed are discarded. The next (in the order of the input file) point is then added to the "seed." This is repeated until all points have either been discarded or are in the seed. The seed is now a list of points that are all at least the “Cutoff” distance away from one another. The "Seed" can be any MATLAB style vector. "1" is the default, but you could use "1:10" or "1:5:100" or "[7,13,52:7:101,218]" all of which describe valid MATLAB vectors.

This method helps to keep the distribution of points somewhat uniform, within the volume of space that the original set occupied. Coarse-graining along the sequence of a protein, keeping every fifth residue for example, will likely result in somewhat non-uniform spatial sampling – possibly a defective model. Since packing density and the shape of the biomolecule are strong determinates of their motions, it is often preferable to coarse grain spatially rather than along the sequence.

2.4) Mixed resolution modeling

One powerful tool in ENM analysis is the ability to run a mixed resolution system. What we mean by mixed resolution is that part of the system is modeled in high detail (say all heavy non-hydrogen atoms) while the rest of the system is modeled in a less detail (say only the alpha carbon atoms). To run such a model, construct a PDB file where the lower resolution (coarser) atoms are listed first and the higher resolution atoms are listed after (see Figure 2.4*i*). The scripts here can help you to build these two components, but familiarity with a molecular viewer may be required.

Select the completed mixed resolution PDB file as your [x,y,z] file and "ANM_mixed" as the ENM type. The input parameter should then be three numbers, each separated by a coma. i.e. "100,13,7" would indicate there are 100 atoms listed in the less detailed (lower resolution) section, a cutoff radius of 13Å for the lower resolution section, and 7Å for the more detailed section.

In Figure 2.4*i* we show an image of the mixed resolution system. The PDB file used in this example is located in the "examples" folder of the MAVEN distribution and named "1T3R_mixedModel.pdb" and is also available on our web page.

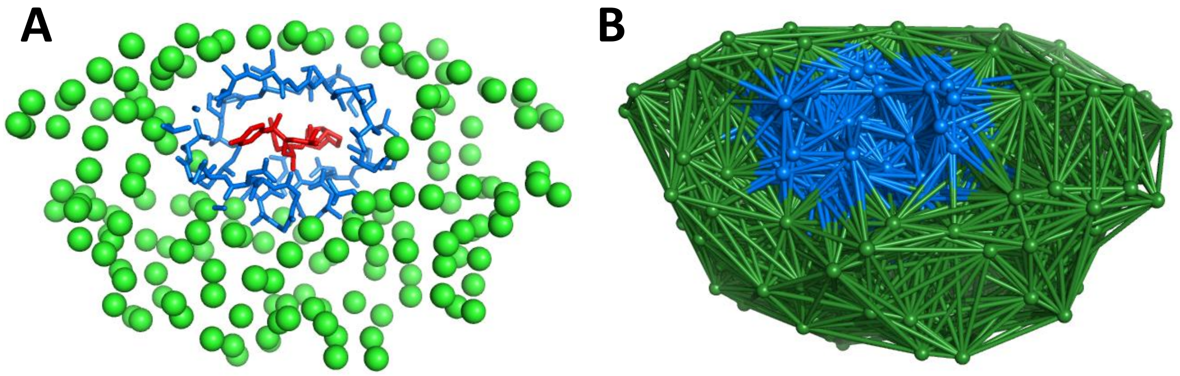

Figure 2.4*i*: A) HIV Protease structure 1T3R with protease inhibitor TMC114 in red and protease atoms within 7Å of the ligand in blue. The remainder of the structure is represented by C^{α} atoms and colored green. B) The elastic network is exported to PyMOL. The default view colors the coarse-grained section green and fine-grained blue.

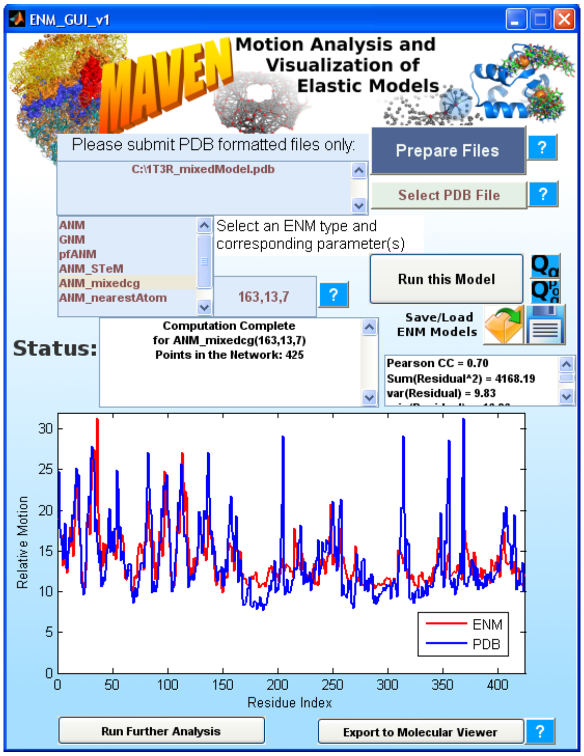

Figure 2.4*ii* MAVEN Interface for Mixed Models
Loading the model into MAVEN, we select an ENM type of "ANM_mixedcg" and set the parameters to "163,13,7" to tell MAVEN that we have 163 coarse-grained points, we want to connect them with harmonic springs if they are within 13Å of each other, and that points within the fine-grained section should be connected if they are within 7Å. Note that the cutoff length that bridges the two is $\sqrt{13 * 7} \approx$ 9.5Å. The result is a model with much better agreement with the experimental temperature factors; correlation coefficient is 0.7. The correlation between experimental and computed B-factors is 0.57 for only alpha carbon coordinates and a cutoff of 13Å. Recalling that the first 163 atoms in the file are for the low resolution section, we can see from the plot on the left that this region in particular has excellent agreement with the experimental data.

3) Running an ENM

One must select an input PDB file, ENM type, and proper parameters to run an ENM model. Help files for each ENM type are available within MAVEN by pressing the ? button with the ENM type selected. Alternatively, quick ENM buttons exist that allow the user to select an unprocessed PDB file and directly run an alpha carbon model.

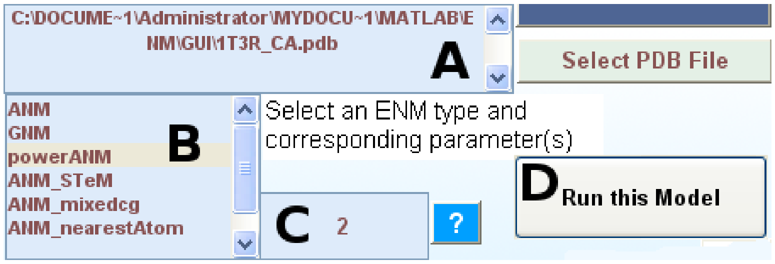

Figure 3.0 Instructing MAVEN what ENM to use. A) Pressing the "Select PDB File" button will bring up a file selection dialog. Navigate to the directory where your PDB file of choice is located and select it. The panel to the left will be updated to show the selected file and full path. If a 4 character PDB structure ID is typed in the text box, then MAVEN will contact the PDB and retrieve the file. B) A list of implemented ENM types. "powerANM" is currently selected. This is the model type that implements distance dependent springs. By clicking the ? button that is visible above, a help file explaining the currently selected ENM type as well as its parameters is displayed. C) Parameter input. For this model, this parameter sets the distance dependence. Thus, points i and j will be connected by springs of strength d_{ij}^{-2}. D) This button tells MAVEN to run the ENM defined by 1) the selected file which contains the points to use, 2) ENM type, and 3) its parameters.

3.1) Quick ENM buttons

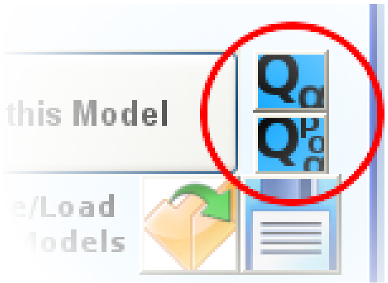

Section 1.3 shows the MAVEN interface and points out two quick ENM buttons which are highlighted to the left with a red circle. The buttons Q_α and $Q_{PO\alpha}$ combine the "Parse CA Model" and "Parse PO4 Model" that are within the Prepare Files module with the "Run This Model" button on the main interface. The result is that one can select an unprocessed PDB file, press the Q_α ($Q_{PO\alpha}$) button, and compute a standard alpha carbon (phosphate, O4', and alpha carbon) model. Thus, these two buttons facilitate the ease with which these models can be computed for proteins alone or for proteins together with DNA or RNA.

One should exercise some caution when using this "quick" feature on diverse PDB structures as there may be more atoms in a given file with Atom Name "CA" than the alpha carbons of the protein (similarly for nucleotide atom types). A good example is that the $Q_{PO\alpha}$ will likely retain phosphate atoms positions from any additional phosphate ions in the solvent or from ATP or ADP. Inspection of generated PDB files will show which atoms have been used.

3.2) Methods and parameter inputs for each ENM type

A list of the ENM types available and their parameters are provided below. Note that parameters are separated by “,” and not whitespace. If a parameter is a vector, then spaces should separate the components of the vector. i.e. [1 1 2 5] is a valid vector, but [1,1,2,5] will be interpreted as 4 parameters, not one.

Most ENM types allow the user to designate the number of modes to solve for. We recommend not asking for fewer than 50 as some of our numerical tests have resulted in poor convergence with fewer modes. The main Status window will alert the user if the eigenvalues do not converge. If this happens, the number of requested modes should be increased.

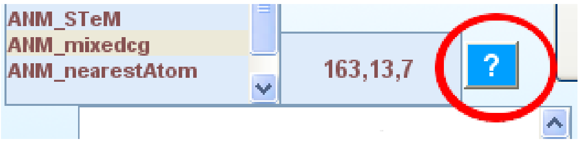

Figure 3.2 Help within MAVEN. The text of section 3.2 is available from within MAVEN by selecting the given ENM type and then pressing the 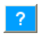

button next to the parameter input box.

3.2.1) GNM - Gaussian Network Model

This function computes the Gaussian Network Model for a given set of coordinates. Any points within a cutoff value are connected by uniform harmonic springs. GNM returns amplitudes of motion within each mode, but no directional information. See Bahar, et. al. (2) for more information.

PARAMETERS:

1) cutoff	Any two coordinates within 'cutoff' will be connected (we suggest a value between 7 and 10Å for alpha carbon models)

3.2.2) ANM - Anisotropic Network Model

An ANM model is generated from a list of points and a cutoff value. Any points within a cutoff value are connected by uniform harmonic springs. A basis set of motions for this system is then computed. Each vector in this basis set is a Normal Mode. Thus, ANM returns directions and relative magnitudes of motion. For more information, see Atilgan, et. al. (1) for more information.

PARAMETERS:

1) cutoff	Any two coordinates within 'cutoff' will be connected (we suggest a value between 11 and 15 for alpha carbon models)
2) numeigs	(optional) number of eigen-pairs to solve for – default solves for all

Example: Cutoff set to 13Å and MAVEN will only compute the first 50 modes: 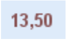

3.2.3) Distance dependent springs - powerANM

We consider all pairs to be in contact, weighted by a power of the distance between them. This function performs an 'all-pairs' ENM where spring strength is dependent upon the inverse power of the distance between nodes.

PARAMETERS:

1) power — distance dependence - spring constants are: r_{ij}^{-power} where r_{ij} is the Euclidean distance between nodes i and j. We suggest a power of 2 for proteins.
2) numeigs — (optional) number of eigen-pairs to solve for – default solves for all

Example: Generating 100 modes with spring strength set to r_{ij}^{-2}: 2,100

NOTE: Rather than the parameter for the cutoff distance, we now have a parameter for the power dependence. For more details including a performance comparison with cutoff based ANM, see Yang, e. al. (4)

3.2.4) STeM – Spring Tensor Model

This is an ANM model that accounts for three and four body interactions; a Spring Tensor Model. See the recent paper by Lin and Song (3).

PARAMETERS:

1) neigen — number of eigen-pairs to solve for. We recommend solving for 50 or more.
2) weight — (optional) the weights used to combine the 4 hessians (bonded pairs, bond angles, tortion angles, and nonboned interactions). Default is [1 1 1 1]

NOTE: Only use spaces for separating the weights. Commas are used for separating parameters.

Example: Running STeM with 100 modes, with changes to bond length and angles weighted twice as strongly as nonbonded, and torsion angle changes weighted by ten: 100,[2 2 10 1]

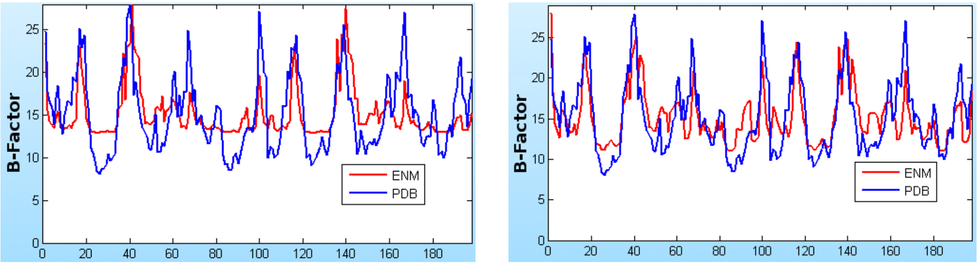

Figure 3.2.4 Effect on STeM performance with parameter choice. (left) Comparison between B-factors deposited for C^{α} atoms in 1T3R and a STeM model with default weights and 50 computed modes. The correlation coefficient is 0.66. (right) We increase the number of computed modes to 100 and change the weights to [2 2 10 1]. These two changes together increase the correlation to 0.74 in addition to a noticeable decrease in the magnitude of the residuals (difference between the curves).

3.2.5) ANM_nearestAtom

This ANM model is much like the standard ANM, but the connectivity matrix is formed from an all atom model. When choosing the PDB input file, select a file with (for example) all heavy atoms in it. ANM_nearestAtom will then extract the alpha carbons and phosphate atoms and connect them with uniform springs if any member of their residue or base is within the given cutoff. If the third input is given an argument that evaluates to True (“1” is fine), then the spring strength is set to the number of atom-atom contacts that fall within the cutoff.

PARAMETERS:

1) cutoff	two coarse-grained points are considered in contact if any atom in their residue or base is within 'cutoff' distance (Å)
2) numeigs	(optional) number of eigen-pairs to solve for – default solves for all
3) weighted	(optional) If true (any positive non-zero number such as “1”), the spring strength equals the number of atom-atom contacts between cg points. If false (“0”), then all springs have a stiffness of 1 (unweighted). The default is unweighted.

3.2.6) ANM with mixed coarse-graining

See Section 2.4 for more information on constructing these models.

One powerful tool in ENM analysis is the ability to run a mixed resolution system. What we mean by mixed resolution is that part of the system is modeled in greater detail (say all heavy non-hydrogen atoms) while the rest of the system is modeled in less detail (say only the alpha carbon atoms). To run such a model, construct a PDB file where the lower resolution (coarser) atoms are listed first and the higher resolution atoms are listed afterwards. The scripts in "Prepare Files" can help build these two components, but familiarity with a molecular viewer may be required. See “Examples\ 1T3R_mixedModel.pdb” in the MAVEN distribution files for an example mixed resolution file.

Select the completed mixed resolution PDB file as your structure file and “ANM_mixed” as the ENM type. The input parameters should then be three numbers, each separated by a coma. i.e. “100,13,7” would indicate that there are 100 atoms listed in the less detailed (coarser) section, a cutoff radius of 13 Angstroms for the lower resolution section, and 7 Angstroms is used for the more detailed section.

Parameters:

1) ncoarse	The number of coarse-grained points in IND
2) coarsecutoff	The cutoff for defining interactions within the coarse-grained points
3) finecutoff	The cutoff for defining interactions within the fine-grained points

*Note that the cutoff used for connecting fine resolution to coarse resolution is the geometric mean of the two cutoff values (square root of their product).

4) Analysis

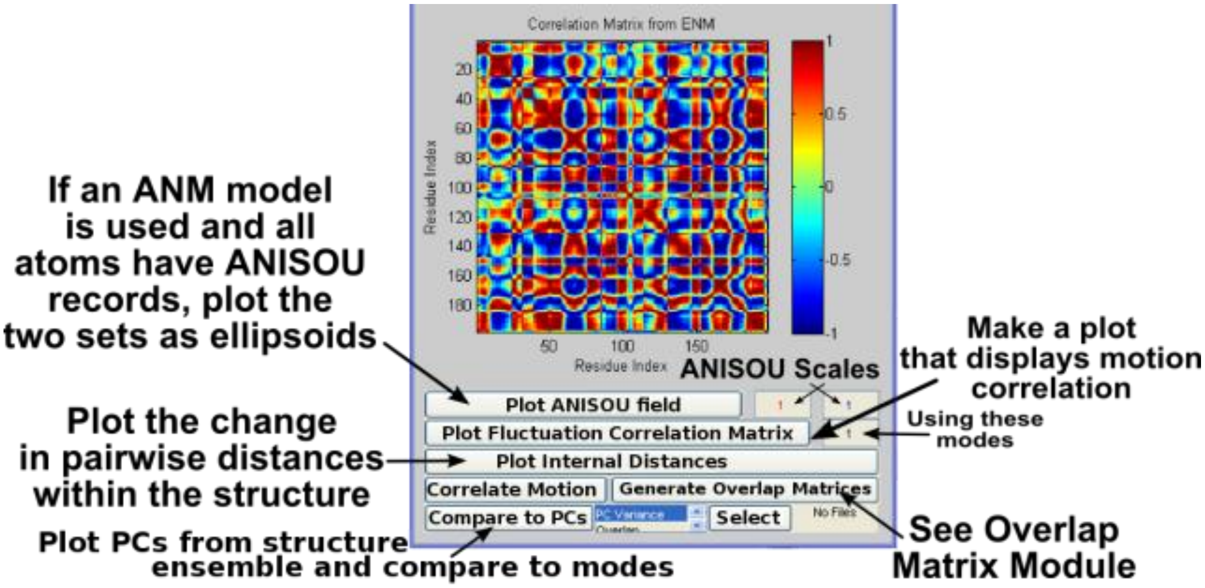

Figure 4.0 Introducing the Analysis module. The analysis capabilities of MAVEN and the user interface. This module is opened from the main MAVEN interface (see Figure 1.3) by pressing the [Run Further Analysis] button. This figure will be referenced in the next subsections.

4.1) B-factors

During X-ray crystallography an electron density is computed for the system. Thermal noise as well as internal motions of the molecule contributes to the “smearing” of this density. Thus, highly mobile atoms may contribute to a relatively large area of electron density. This phenomenon is quantified in a metric known as the Debye-Waller or B-factor and is recorded in the PDB file. Because thermal motions scale with temperature, they are also referred to as temperature factors. It is vital to note that all B-factors can have substantial contributions of rigid body motion, experimental noise, errors, heterogeneity in the crystal, etc; which can all contribute to B-factors. Thus, it is not always clear *a priori* how well the B-factors capture the actual solution dynamics of molecules. After calculating any ENM model, a theoretical B-factor curve is calculated from the ENM and displayed on the main MAVEN interface along with any B-factors in the input structure file (see Figure 1.3).

Understanding which parts of the structure are correlated in the dominant motions can be very informative. The [Plot B-factor Correlation Matrix] button (see Figure 4.0) does this by computing:

$$C(i,j) = \frac{\langle \Delta R_i \cdot \Delta R_j \rangle}{\{\langle \Delta R_i \cdot \Delta R_j \rangle \langle \Delta R_i \cdot \Delta R_j \rangle\}^{1/2}}$$

The C matrix then represents the orientational cross-correlations between the fluctuations of ENM points. Plots of C for individual modes, or a collection of modes will provide information about what parts of the structure move collectively. For a more in-depth look at how specific regions of the structure move with respect to each other see Section 4.2, Overlap Matrices.

The normal mode fluctuations alter the relative position of atoms within the structure. This change in the relative positions of atoms can be quantified by the internal distance changes:

$$\langle (\Delta R_i - \Delta R_j)^2 \rangle = \langle \Delta R_i^2 \rangle + \langle \Delta R_j^2 \rangle - 2 * \langle \Delta R_i * \Delta R_j \rangle$$

These are computed directly from the pseudo inverse constructed as: $H^{-1} = \sum_{i=7}^{c} \frac{1}{\lambda_i} Q_i^T Q_i$, where Q_i is a normal mode, λ_i the corresponding squared frequency, and *c* the largest mode index we wish to consider. The first 6 modes correspond to rigid body translation and rotation of the whole system, and so their contribution is neglected.

4.2 Anisotropy of Motion

This function is accessed by pressing the 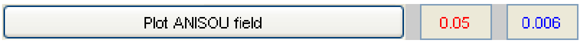

button. To the right of this button are two numbers. They are the scaling factors used for the two sets. If the numbers are changed and the button pressed again, then the ellipsoids will be redrawn with the new scaling.

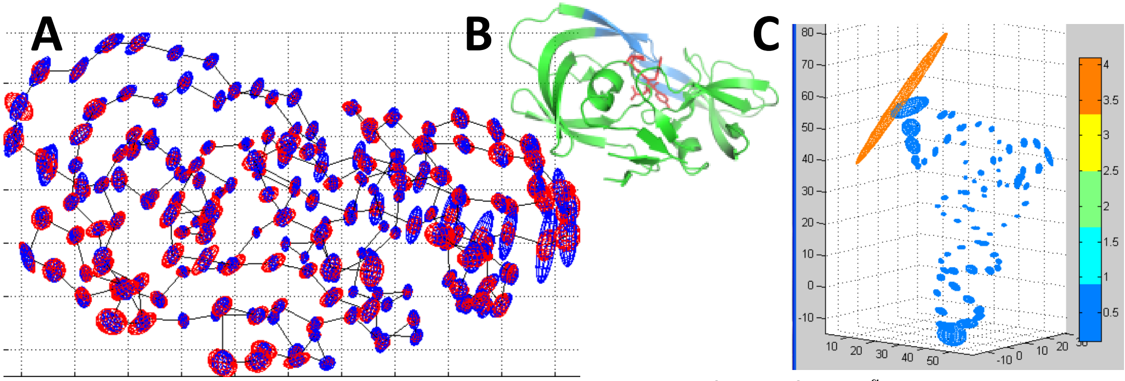

Figure 4.2 Visualizing Anisotropy A) Anisotropic temperature factors from C$^{\alpha}$ atoms in 1T3R are shown as red mesh ellipsoids. From ANM models, the concept of mean square fluctuations generalizes to a 3-by3 tensor that describes more details of the directions of distortions. Anisotropy of motion is more difficult to model, but may be important for function. Always keep in mind what the experimental data is, however. If anisotropies are strongly influenced by crystal packing, then low agreement with the ANM results is not necessarily unwanted. A similar metric can be derived from NMR ensembles by calculating the variance of each atom across the models. B) We show a cartoon representation of the protein to show the orientation seen in A); inhibitor colored red and active site flaps blue. This orientation was chosen to emphasize the differences in anisotropy between the ANM and ANISOU records in the PDB file. C) In the case of this tRNA structure (1TRA), no experimental ANISOU records were found. When this is the case, MAVEN will plot the ANM-derived fluctuations and color them by their relative volume. We see that the terminal base on the acceptor arm dominates the motion (the so-called “tip effect” which is usually lessened with the nearest atom or STeM methods).

4.3) Fluctuations in internal distances

ENMs allow us to efficiently compute not only mean square fluctuations, but also fluctuations in internal distances. This quantity describes the average fluctuation in the distance between atom *i* and atom *j* within the modes and is calculated using:

$$< (\Delta R_i - \Delta R_j)^2 > = (3k_B T/\gamma) * [\Gamma_{ii}^{-1} + \Gamma_{jj}^{-1} - 2\Gamma_{ij}^{-1}]$$

where k_B is the Boltzmann constant, γ the spring constant between atom *i* and *j*, and , $\Gamma^{-1} = \sum \frac{1}{\lambda_i} Q_i Q_i^T$, where the summation of normal modes, Q_i, and square frequencies, λ_i (the eigenvector and eigenvalues,

respectively). We compute the pseudo-inverse because Γ has zero-value eigenvalues and is by definition not invertible and requires the use of singular value decomposition.

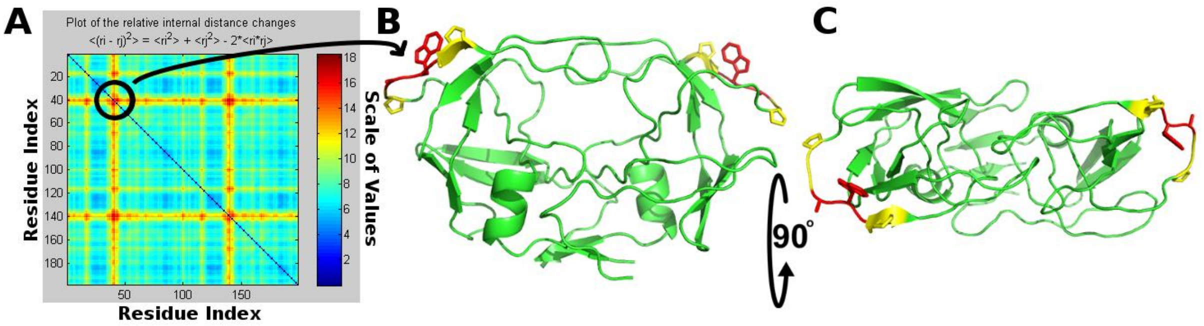

Figure 4.3 Visualizing changing internal distances by using the [Plot Internal Distances] button (see Figure 4.0). We compute an alpha carbon ANM model and visualize the internal distance change matrix using all modes in MAVEN. The structure is visualized in PyMOL with residues colored by the alpha carbon changes. A) The internal distance changes upon mode deformation are computed from the above equation and visualized within MAVEN. An arrow relates one of the two largest areas of change to the structure. B) The six residues in each chain with target changes are colored and side chains displayed. C) Rotated view.

4.4) Correlated Motion between two parts of the structure

Determining what the normal modes mean for the structure is not always a straightforward task and analyzing the motion of subsets of the structure can be instructive. We have developed an analysis module that compares how two parts (subsets of the structure - groups) move in one or a few modes in order to better understand what the mode-motion means. Note that this type of analysis is, in some ways, a simplification of the Overlap Matrix module described in section 4.6, but may be more meaningful for looking at the relationship between two groups, as opposed to a larger number of groups. This type of analysis is accessible using the **Correlate Motion** button, visible in Figure 4.0. In Figure 4.4.1 we show an example of comparing the two termini of 1T3R. There are 198 C^{α} atoms in this structure and as such, the atoms are indexed from 1 to 198. Thus, the N-terminal ten C^{α}s are chosen with the syntax 1:10 and the ten C-terminal with 189:198. We choose modes 1 through 5 (1:5) for the comparison. When more than one mode is chosen, an overlap matrix is constructed using the first selected mode (1 in this case) which consists of a heatmap of pairwise dot products between the direction of each atoms motion in the given mode. A second plot is made where the Mean Square Fluctuation (MSF) from ENM using only the selected modes (here, 1 through 5) is plotted with the experimental B-factors from the PDB file for both groups. Any status changes including errors thrown due to improper parameter syntax will be shown in the main status window (see Figure 1.3).

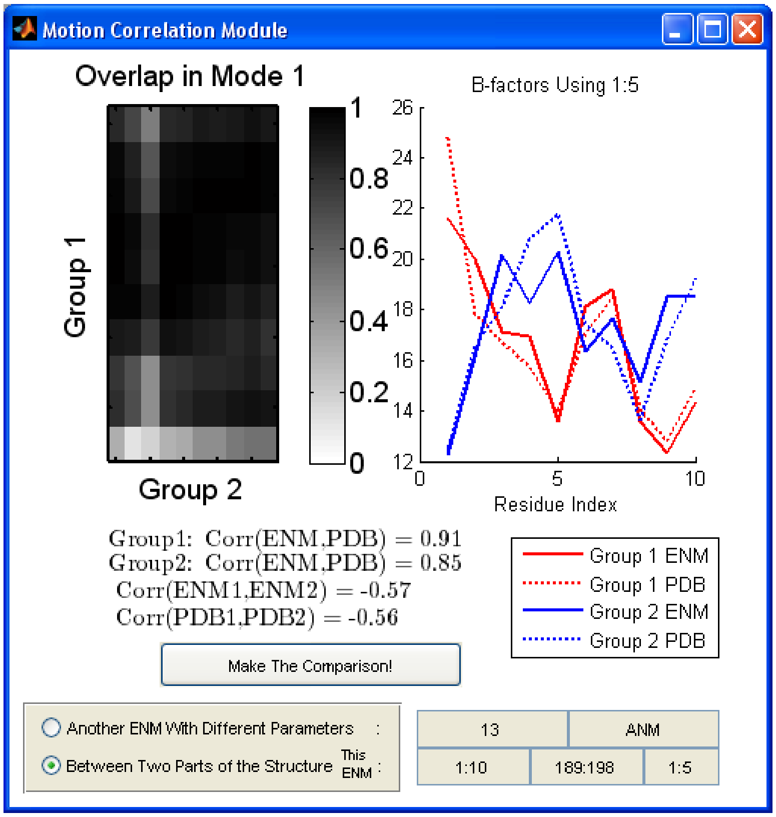

Figure 4.4.1 Comparing Motion Within an ENM. Here we show a comparison between the motion of the N- and C-terminus of 1T3R using only the first mode (Overlap) and the first 5 modes (MSF calculated using 1:5). Here we have used an ANM model with a cutoff of 13Å. We see that the two termini are well correlated with each other in the first mode and that the MSF from ENMs is very similar to the B-factors from X-ray crystallography. Pearson's correlation coefficients are displayed between the MSF and experimental (PDB) values. When the two parts of the structure (groups) are of the same size, the correlation between groups is also displayed.

4.5) Compare one ENM to Another

Another useful feature of MAVEN is the ability to compare one ENM to another. This dialog is initiated using the Correlate Motion button, visible in Figure 4.0. To begin, we show a comparison between linear distance dependent springs and quadratic distance dependence in Figure 4.5.1. The overall motion is not changed, but there is a shuffling of the first 6 low-frequency modes between the two models. This type of analysis can be used to help researchers decide what the effect of various ENM types are on their system. The Cumulative Overlap is also shown. This quantity captures the total overlap between 3, 6, 10, or 20 modes in the initial ENM model, and a combination of modes from the new ENM whose parameters are designated below. Note also that GNM does not contain directions of motion, so this type of analysis is of less value for comparing to GNM. Any status changes including errors thrown due to improper parameter syntax will be shown in the main status window (see Figure 1.3).

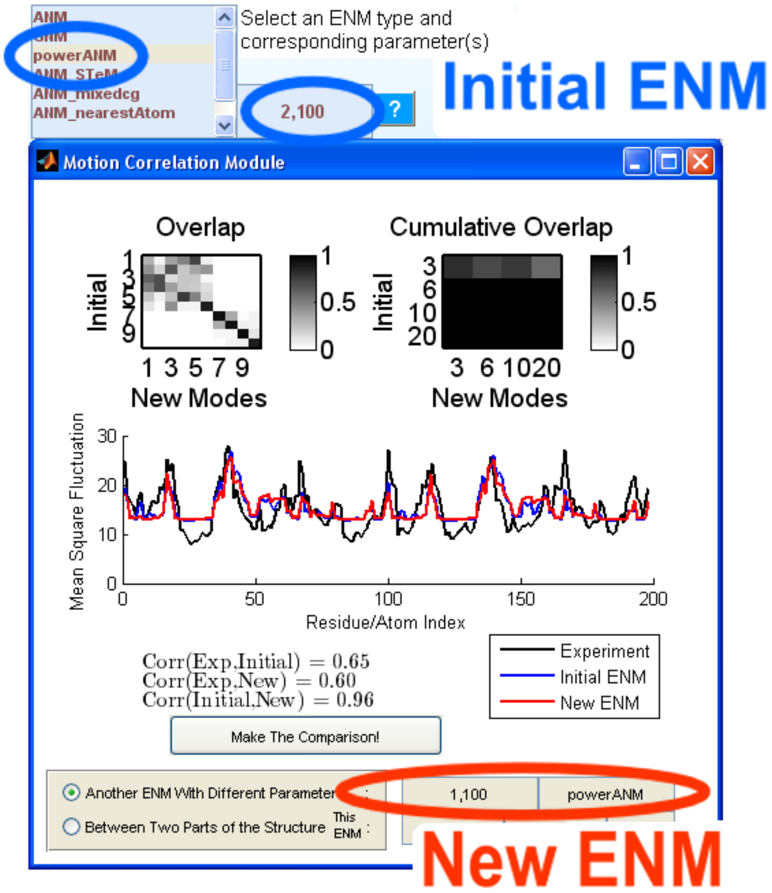

Figure 4.5.1 Comparing one ENM to another – New Parameters. Here we are comparing the distance dependent springs model with a power dependence of 2 (using 1T3R) with a linear power dependence – in both cases, 100 modes are calculated. We find that the mobility of each atom is not significantly changed for this structure (the MSF curves are almost superimposed), but that there has been a shuffling of the first 6 modes (Overlap matrix). Pearson's correlation with the experimental values recorded in the PDB file are also displayed.

Changing the parameters used can have a large effect on the model, but using a different ENM type may be a more interesting comparison. The Motion Correlation Module also has the ability to perform this type of comparison. In Figure 4.5.2 we compare a cutoff based ANM to a distance dependent springs model. The "initial model" is the ENM model what is generated from the main MAVEN interface. The parameters and ENM type for the new model (to which the initial is compared) are specified within the Motion Correlation Module (see Figure 4.5.2).

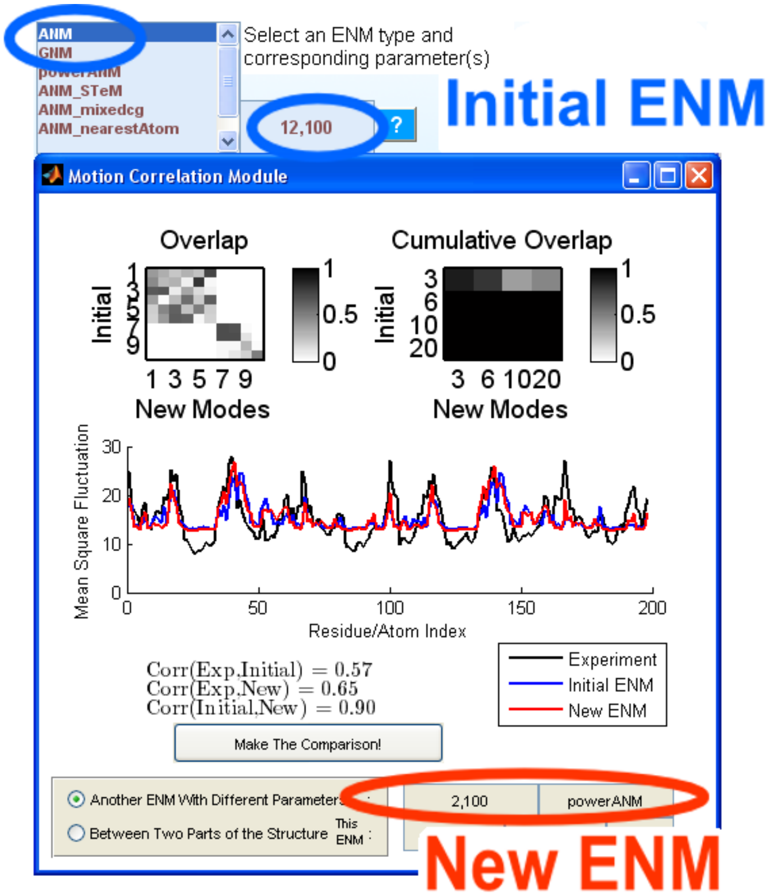

Figure 4.5.2 Comparing one ENM to another – New ENM Type. In this case, we are comparing ANMs of two different types. We compare a cutoff based ANM using a cutoff of 12Å with a distance dependent model and a distance dependence of 2. In both cases we use the structure 1T3R and 100 modes are calculated. Again, we do not find a large difference in the patter of fluctuations, but the extent of overlap between modes from each model has decreased. The distance dependent springs do offer an improved agreement, which is indicated by an improvement in the B-factor comparison – the correlation increases from 0.57 to 0.65.

4.6) Overlap Matrices → Correlated motion within the structure

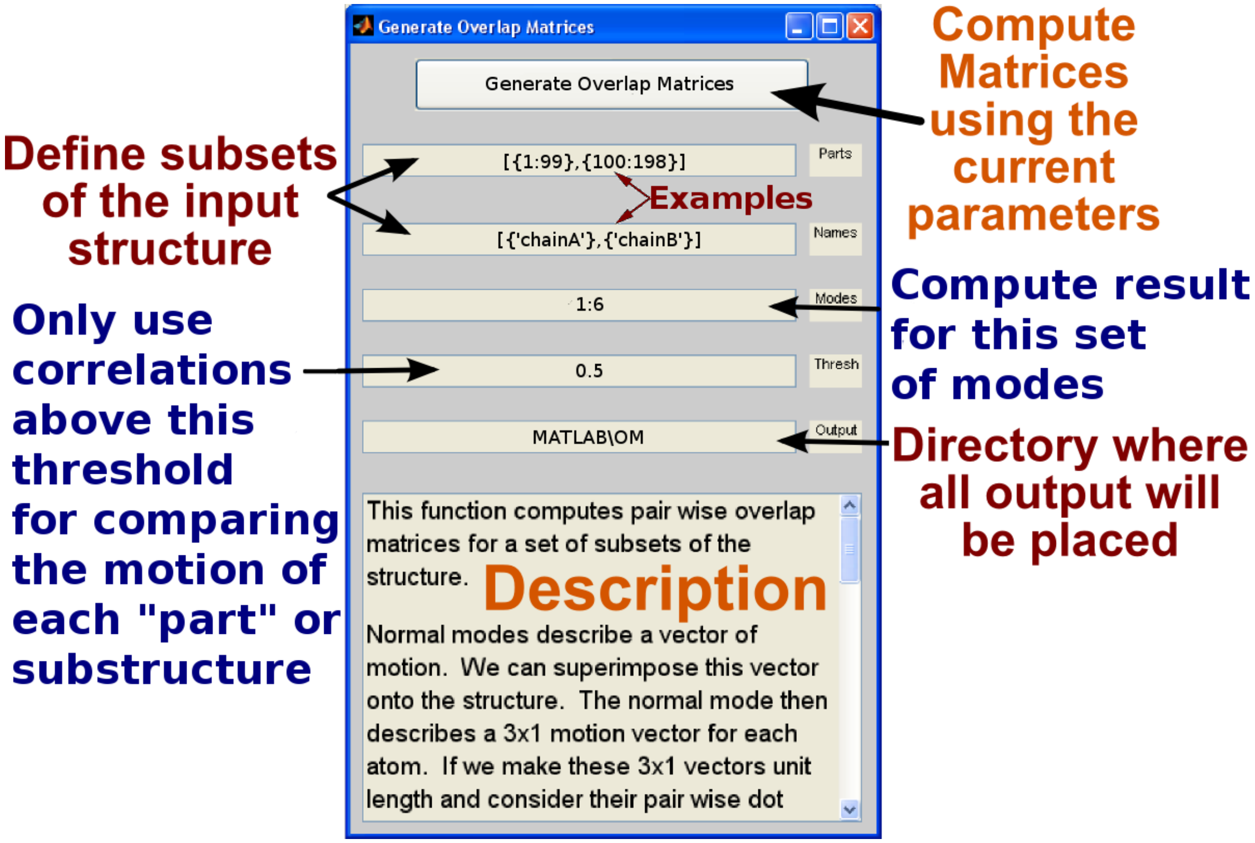

Figure 4.6 Schematic of the Overlap Matrix dialog for computing directional correlations of computed motions within the structure. This function computes pairwise overlap matrices for the specified modes and parts (or subsets) of the structure. Access this module by pressing the [Generate Overlap Matrices] button in the Analysis module (see Figure 4.0). Normal modes describe a vector of motion which we can superimpose onto the structure. The normal mode then describes a 3x1 motion vector for each atom. If we make these 3x1 vectors unit length and consider their pairwise dot product, we will end up with a matrix describing the amount of motion in the same direction, opposite direction, or orthogonal. Matrices of this type are termed “overlap matrices.”

NOTE: As the number of “parts” and requested modes increases, the algorithm may generate a large number of images. There will be one image made that compare motion within each mode and for each of the pairwise “parts.” In addition to these, summary matrices will be made, one for each mode, that display the dominant trends. See Figure 4.6.2 for a specific example. It is recommended that MAVEN not be interrupted while the computation is in progress. Doing so has the potential to disrupt the image saving script.

Defining the regions (Parts) within the structure that we wish to follow takes on MATLAB cell array syntax where each cell describes one region. The description of a region is a vector of node indices. By node indices, we mean the index of the node or geometric point as it is read into MAVEN. If records other than ATOM and HETATM have been trimmed from the PDB file, then there will be a one-to-one correspondence between node index and PDB line number. Depending on how the input PDB file was generated, this many not necessarily correspond to the atom numbering.

A directory for the output must be given. This module will generate a folder of data for each mode of motion containing comparisons of all unique pair-wise combinations of the various “Parts.”

EXPLANATION OF VARIABLES
EXAMPLES ARE FOR 1T3R.pdb

part = {}

- cell array where cells are vectors describing the subsets to use.
- each cell should be a vector of induces from the parent structure
- i.e. [{1:99},{100:198}]
  → 1T3R is a homodimer. Our first example (figure 4.6.1) simply compares the dimmers.

names = {}

- cell array of names of the subsets
- each cell is a string that names the corresponding "part"
- i.e. [{'chainA'},{'chainB'}]
  → 1T3R is a dimer. We label each part by its PDB chain (see figure4.6.1)

Modes = 1:6

- which normal modes to consider
- the contribution of each mode to the total motion diminishes quickly as the mode index increases
- between 6 and 20 modes are suggested for this type of analysis

threshold = 0.5 (±)

- only overlaps above this magnitude will be counted in the summary matrices, for both positive and negative correlations

outdir → the output directory

- directory where the output is deposited
- subdirectories will be made for each mode and all unique overlap matrices placed therein
- each mode will also be summarized in two heatmaps (see Figure 4.6.2)

Example 1: HIV Protease 1T3R
Parts: [{1:99},{100:198}]
Names: [{'chainA'},{'chainB'}]

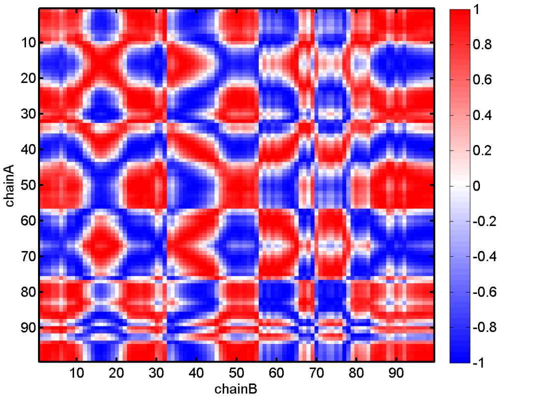

Figure 4.6.1 Example of overlap matrix. The result of this computation is a set of images and data files placed in the output directory. This type of matrix will be referred to as an Overlap Matrix and each point in the heatmap is the overlap (dot product) between the directions of motion that each point moves for the given mode (mode 1 shown). A value of 1 means that the two points move parallel to each other in the mode, and -1 is motions in opposite directions or anti-correlation of motion.

Next, we consider eight subsections of the structure. The HIV-1 Protease is a homo dimer (the two monomers are referred to as chain A and chain B) where each monomer contains a “flap” domain that covers the substrate. A “shoulder” attaches this flap to the bulk of the structure (core). The N-Terminus can also be considered as a unit. We compute overlap matrices for all unique combinations of these eight (four per monomer) subsets of structure in the following.

Parts: [{1:5},{44:58},{[36:43 59:62]},{[12:35 44:58 63:93]},
{100:104},{143:157},{[135:142 158:161]},{[111:134 143:157 162:192]}]

Names: [{'TermA'},{'FlapA'},{'shoulderA'},{'CoreA'},
{'TermB'},{'FlapB'},{'shoulderB'},{'CoreB'}]

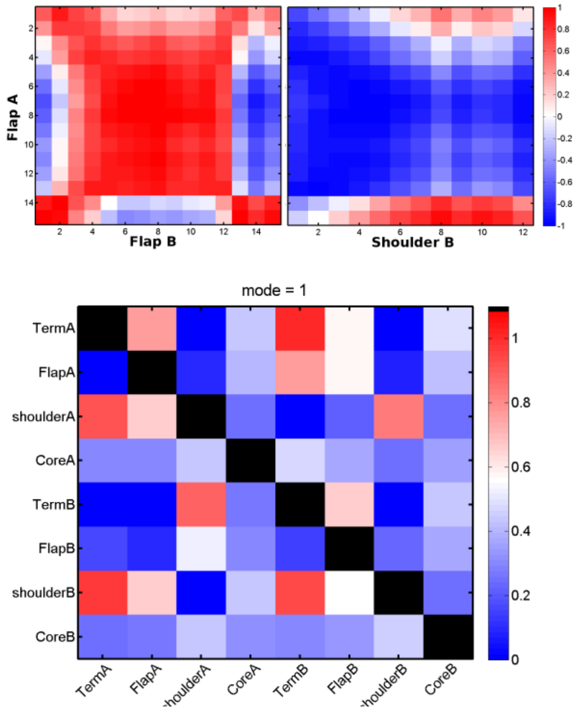

Figure 4.6.2 Heatmaps describing the details of motion direction within the first mode of motion. With a larger number of subsections of the structure defined (above), we can better appreciate the dynamics of the structure. Two example overlap matrices are shown on the left. From these matrices we see that atoms in the flaps move in strong positive correlations with each other, while flap A and shoulder B are almost totally anti-correlated. For each mode, images of this type will be made for all unique pairwise combinations of the Parts. **Matrices which summarize all the overlap matrices for each mode are also generated.** The first type (visible to the left), has the fraction of each overlap matrix that is above the threshold value (0.5 used here) and positively correlated in the upper triangle, while the lower triangle displays the fraction of values within each overlap matrix whose magnitude is greater than the threshold and are anti-correlated. To relate the above two overlap matrices to this summary matrix, we consider the “Flap A” row, move across to the “Flap B” column, and see a white square. By the colorbar we see that about half of the values in the “FlapA-FlapB” overlap matrix are positive and greater than 0.5. We also find information such as (1) the termini exhibit the strongest positive overlap (2) both termini have strong negative overlap with both shoulders (3) the cores are the largest subsection and have little discernable overlap pattern with any other part of the structure in this mode of motion. The other type of summary matrix (not shown here) displays

only an upper triangular matrix and records the fraction of each overlap matrix that has a magnitude greater than the threshold (absolute value of the overlap), by combining correlations and anticorrelations. By choosing appropriate subsets of points, detailed information about the mode shapes can be determined.

4.7) Compare modes to Principal Components of a structural ensemble

The goal of most ENM studies is to determine the dominant functional motions of biomolecules. With the ever growing numbers of experimentally solved structures, homology modeling, and molecular simulation, gathering a picture of the functional ensemble of conformations of a biomolecule is increasingly a tractable problem. All three methods can either store the conformers as separate PDB files, or as one multi-MODEL file. Both types of storage are supported by MAVEN.

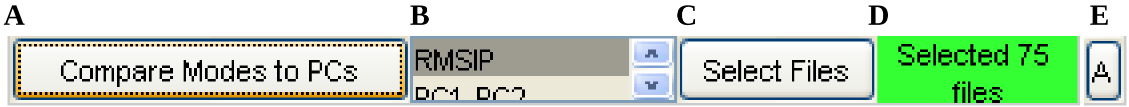

Figure 4.7.1 Explanation of PC construction. A) When this button is pressed, the plot type that is selected will be generated and displayed. B) Select a plot type. The options include the variance in each PC, the dot product between modes and PCs (Overlap), the Cumulative Overlap, Root Mean Squared Inner Product (the percent of the PC spanning space that is shared by the normal modes), and PC plots. The PC plots make a scatter plot where each structure in the ensemble is a point. C) Pressing this button will open a "file select" dialog. The user should select all PDB files that they wish to include in the structural ensemble, from which the PCs will be constructed. These may be PDB files for one conformation or multi-MODEL files. One could even save frames from an MD trajectory in PDB format and use them here. D) After processing the files and finding them to contain the same number of points as the current ENM model, the number of conformers is displayed. E) This button helps the user to annotate PC plots. Pressing it will turn the cursor into a selection crosshair. When points in the plot are clicked on with the crosshair, up to the first 8 letters of its file name are displayed next to the point.

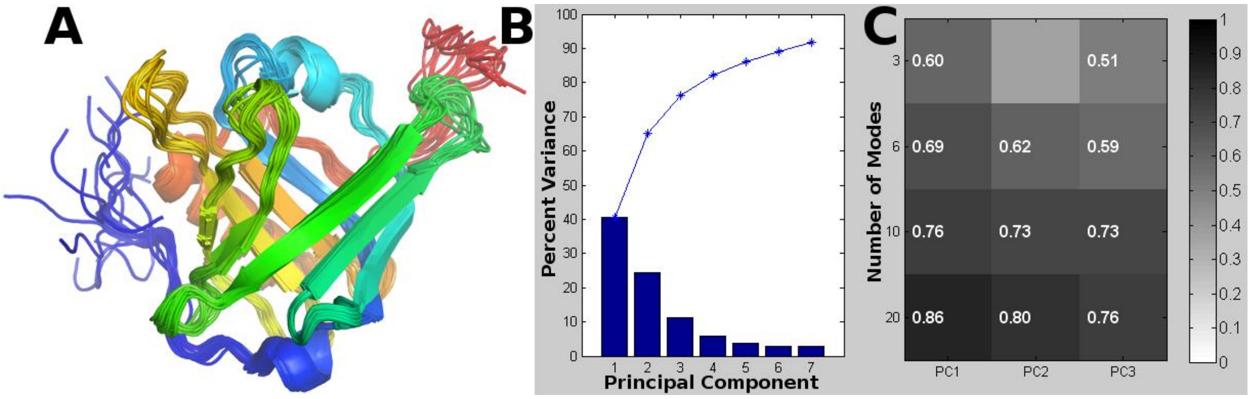

Figure 4.7.2 PCA analysis of 2KTD. A) Visualizing the structural ensemble of in PyMOL – coloring is blue-to-red from the N-terminus to C-terminus. There are 15 models in the ensemble. B) MAVEN plot showing the percent of ensemble variance that is captured by each PC as well as the cumulative. C) MAVEN plot showing the cumulative overlap (dot product) of the first 3, 6, 10, or 20 modes with each of the first 3 PCs. In this case we have generated 200 modes from the STeM ANM model that accounts for changes in bond length, bond angle, and torsion angle.

5) Exporting to Text or Molecular Viewers or Text Files

Often it is useful to visualize the normal modes moving the structure, or to save them for other uses. Both of these options are available within MAVEN from the Export module. Access this module by pressing the [Export to Text or Molecular Viewer] button on the MAVEN main interface (see Figure 1.3).

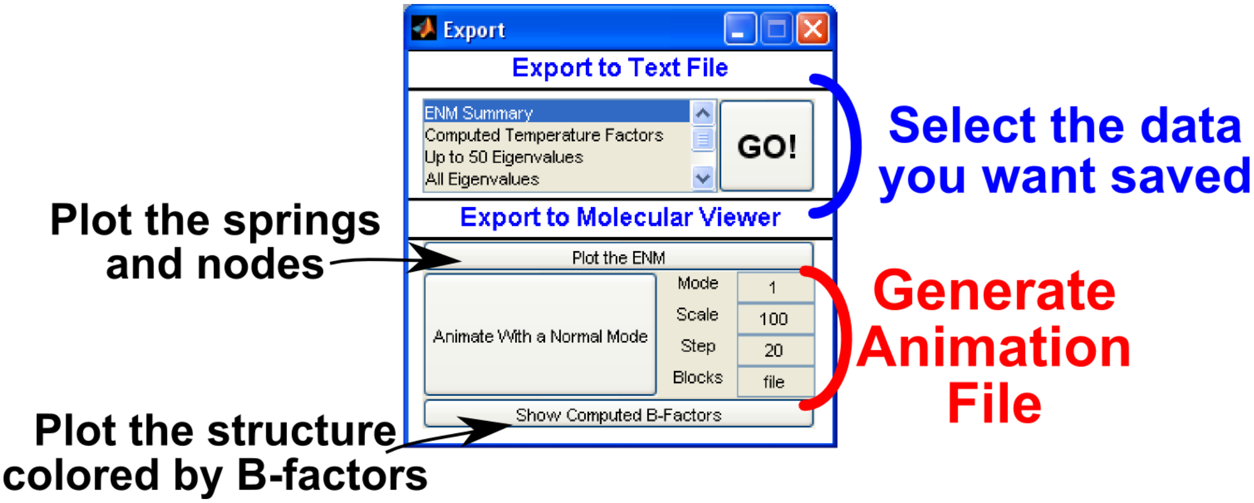

Figure 5.0 The Export Module Menue. Two main export features exist: plain text and PDB file. Text files can be generated containing information about the ENM that was run (type, parameters, etc), the eigenvalues, or eigenvectors. [Plot the ENM] will make a PDB file where bonds are explicitly added that correspond to the springs used in the ENM. Animations take the form of a multi-MODEL PDB file where each MODEL describes one frame (or state). If "Blocks" is set to a file name, then a one-to-one mapping from ENM point to residues in the block file will be assumed. The residues (determined by residue ID) will then move as rigid blocks based on the motion of the corresponding ENM point.

5.1) Exporting Data to a Text File

Select which data types you would like to export to a text file. The [ctrl] and [Shift] keys can be used to select multiple options. The first six eigenvalues and eigenvectors are ignored since they represent the degrees of freedom for rigid body rotation and translation. Note that the eigenvectors (normal modes) are printed to the file in a comma separated format and each line has up to six numbers. Each mode is then the concatenation of these rows.

Mode: 1
value1,value2,value3,value4,value5,value6
value7,…,value12

5.2) Exporting the ENM model to a molecular viewer

This module provides options for exporting the active ENM model to a molecular viewer. Which viewer (PyMOL, VMD, etc.) will be used and how the representations will appear when initially loaded is defined in a configuration file. See MAVEN_Export.conf on our web page for examples of these configuration files.

The model can be made into an animation. "Scale" is a scalar value describing the maximum amount of deformation along the requested mode. This is usually chosen to be sufficiently large for viewing purposes. The default value of 100 is a good starting point. The main Status window (see figure 1.3) will report maximum RMSD in the animation. "Step" defines the step size that each frame (also the MODEL number in the resulting PDB file) will take. If "Block" is a file name, then this file is read and then the "blocks" are animated. More precisely, we will assume that each node in the ENM corresponds to one residue in the "Block" file (a PDB file). Each residue will then move rigidly in the same way as its point in the ENM. If one performs alpha carbon coarse-graining, then residues will move rigidly and the animation can be displayed with cartoon representations, rather than simply the alpha carbon trace. Note that the "Block" file must have the same number of residues as there are nodes in the ENM. If not, MAVEN will not know which residues correspond to the modeled points.

Many resources exist for making interesting figures using VMD and PyMOL. Some of them are listed here:

PyMOL wiki:	http://pymolwiki.org/index.php/Main_Page
PyMOL script library:	http://pymolwiki.org/index.php/Category:Script_Library
	http://pldserver1.biochem.queensu.ca/~rlc/work/pymol/
PyMOL community:	http://sourceforge.net/mailarchive/forum.php?forum_name=pymol-users
VMD community:	http://www.ks.uiuc.edu/Research/vmd/mailing_list/vmd-l/

As these software and resources already exist, MAVEN does not need to reproduce them, but should work seamlessly with them. Many scripts, plug-ins, and functions in VMD for trajectory analysis will work with mode animations from MAVEN. Animation functions in most molecular viewers will interpret the multi-MODEL PDB files as frames in an animation. Thus, MAVEN integrates into the already powerful analysis and visual options within these systems.

5.3) Further examples of visualizations of MAVEN output in PyMOL:

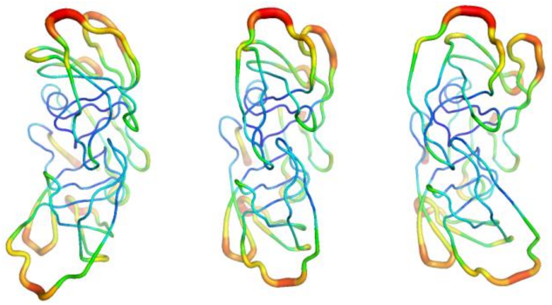

Example 1: (left) the negative direction of mode 1 generated from distance dependent springs with a square dependence and PDB 1T3R. (center) The unaltered structure. (right) the positive direction of the mode and (left) negative direction. Top down view.

Generated with: cartoon putty

Color → Spectrum → B-Factors (CA)
Using "create" to make objects for individual frames
Using grid_mode and grid_slot to show the created objects next to each other

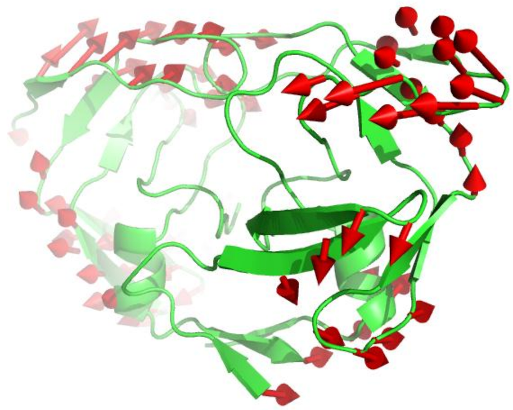

Example 2: We show the initial structure (same ENM model as mode 1) with mode 1 displayed using the modevectors script available on the pymol wiki.

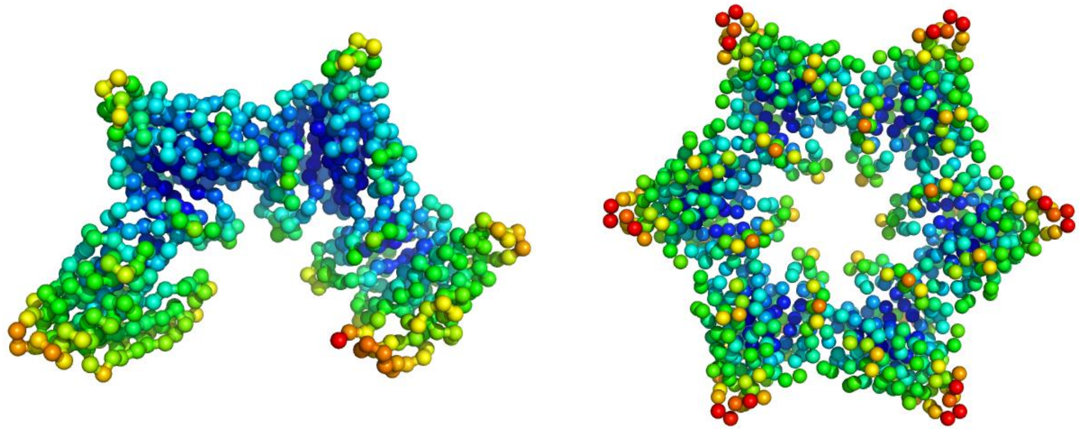

Example 3: Alpha carbon atoms from 1YYF tetramer (left) and hexamer (right) colored by their mean square displacement computed from all ANM modes. This emphasizes the need to have the full structure present when generating biologically meaningful motions. Also, one may interpret the highest mobility of the tetramer as the regions that are "seeking" other binding partners with highest motions until the full structure is formed. Then the motions shift from assembly dynamics to functional dynamics.

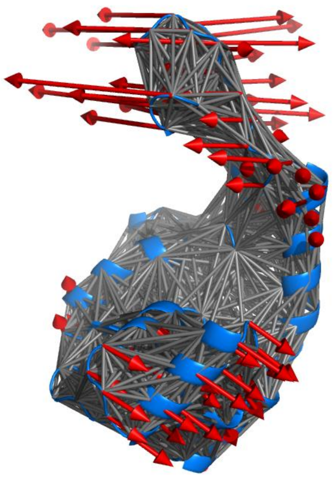

Example 4: Adenylate kinase shown as a cartoon with gray bars indicating the springs from the ANM. Red arrows indicate motions in the first mode – drawn with the modevectors script from the pymol wiki. This is an open form of the structure. The dominant mode describes a motion where the open active site flap (top of the current view) moves down toward the body of the structure, moving towards the closed conformation.

Please also see the MAVEN publication in BMC Bioinformatics

Reference List

1. Atilgan, A. R., S. R. Durell, R. L. Jernigan, M. C. Demirel, O. Keskin, and I. Bahar. 2001. Anisotropy of fluctuation dynamics of proteins with an elastic network model. Biophys. J. 80: 505-515 PM:11159421.

2. Bahar, I., A. R. Atilgan, and B. Erman. 1997. Direct evaluation of thermal fluctuations in proteins using a single-parameter harmonic potential. Fold. Des 2: 173-181 PM:9218955.

3. Lin, T. L., and G. Song. 2010. Generalized spring tensor models for protein fluctuation dynamics and conformation changes. BMC Struct. Biol. 10 Suppl 1: S3 PM:20487510.

4. Yang, L., G. Song, and R. L. Jernigan. 2009. Protein elastic network models and the ranges of cooperativity. Proc. Natl. Acad. Sci. U. S. A 106: 12347-12352 PM:19617554.
